# High-yield production of recombinant platelet factor 4 by harnessing and honing the gram-negative bacterial secretory apparatus

**DOI:** 10.1371/journal.pone.0232661

**Published:** 2020-05-07

**Authors:** Saeed Ataei, Mohammad Naser Taheri, Gholamhossein Tamaddon, Abbas Behzad-Behbahani, Fatemeh Taheri, Amir Rahimi, Farahnaz Zare, Niloofar Amirian

**Affiliations:** 1 Diagnostic Laboratory Sciences and Technology Research Center, School of Paramedical Sciences, Shiraz University of Medical Sciences, Shiraz, Iran; 2 Department of Medical Biotechnology, School of Paramedical Sciences, Shiraz University of Medical Sciences, Shiraz, Iran; 3 Bioinformatics and Computational Biology Research Center, Shiraz University of Medical Sciences, Shiraz, Iran; 4 Department of Mechanical Engineering, McGill University, Montreal, Canada; 5 Department of Molecular Medicine, School of Advanced Medical Sciences and Technologies, Shiraz University of Medical Sciences, Shiraz, Iran; Rutgers University/New Brunswick, UNITED STATES

## Abstract

Platelet factor 4 is a cytokine released into the bloodstream by activated platelets where it plays a pivotal role in etiology and diagnosis of heparin-induced thrombocytopenia. Therefore, a sustainable source of recombinant PF4 with structural and functional similarity to its native form is urgently needed to be used in diagnostic procedures. To this end, a three-in-one primary construct was designed from which three secondary constructs can be derived each capable of employing either type I, type II secretory or cytoplasmic pathways. Protein expression and secretion were performed in *Escherichia coli* BL21 (DE3) and confirmed by SDS-PAGE and Western blotting. To further enhance protein secretion, the effect of several controllable chemical factors including IPTG, Triton X-100, sucrose, and glycine were individually investigated at the outset. In the next step, according to a fractional factorial approach, the synergistic effects of IPTG, Triton X-100, and glycine on secretion were further investigated. To ascertain the structure and function of the secreted recombinant proteins, dynamic light scattering was utilized to confirm the rPF4 tetramerization and heparin-mediated ultra-large complex formation. Moreover, Raman spectroscopy and Western blotting were exploited to evaluate the secondary and quaternary structures, respectively. The type II secretory pathway was proven to be superior to type I in the case of rPF4 secretion. Supplementation with chemical enhancers improved the protein secretion mediated by the Type II system to approximately more than 500 μg/mL. Large quantities of native rPF4 up to 20 mg were purified as the culture medium was scaled up to 40 mL. Western blotting confirmed the formation of dimers and tetramers in the secreted rPF4 proteins. Dynamic light scattering revealed the rPF4 oligomerization into of larger complexes of approximately 100–1200 nm in size following heparin supplementation, implying proper protein folding and tetramerization. Moreover, the rPF4 secondary structure was found to be 43.5% Random coil, 32.5% β-sheet, 18.6% α-helix and 4.9% Turn, which is in perfect agreement with the native structure. Our results indicate that the gram-negative type II bacterial secretory system holds a great promise as a reliable protein production strategy with industrial applications. However, further efforts are required to realize the full potential of secretory pathways regarding their application to proteins with distinct characteristics.

## 1. Introduction

Heparin-induced thrombocytopenia (HIT) is a deleterious drug reaction caused by heparin administration in which platelet factor 4 (PF4) as a positively charged protein binds heparin, and the resulting PF4-Heparin complex adversely stimulates an immune response. Following engagement with the FcγIIa receptors, a PF4-Heparin-IgG complex activates platelets and therefore gives rise to thrombosis. The disease etiology also includes antibody-mediated endothelial trauma or excessive tissue factor production in cases where the antigen-antibody complexes interact with monocytes [1–5]. Currently, there are several approaches to diagnosing patients with HIT, with some of them requiring exogenous PF4 [6,7]. Therefore, a cost-effective, sustainable, and scalable source of functional recombinant PF4 is imperative.

PF4 is a 7.8 kDa protein, consisting of 70 amino acids in its mature form and possesses two disulfide bonds. [8] It is released during platelet activation and plays a plethora of biological roles including regulation of angiogenesis, megakaryopoiesis, and activation or proliferation of leukocytes [9–12].

Protein secretion is a pivotal biological phenomenon in survival and pathogenesis of many bacteria. Hitherto, gram-negative bacteria are known to have at least six distinct protein secretory pathways. Type I and II have been mainly used for recombinant protein production. [13]

The protein substrates of type I secretion system (T1SS) are characterized by a non-cleavable C-terminal signal sequence recognized by the components of the T1SS machinery and are directly secreted to the extracellular milieu. T1SS protein machinery is composed of HlyB, an ATP-binding cassette (ABC) transporter, and HlyD, a protein that connects the inner membrane to the outer membrane. The T1SS system is epitomized by the HlyA protein of the pathogenic *E*. *coli* [14–16].

The type II secretion system (T2SS) has been found to be a well-suited pathway for the production of some recombinant proteins. In this system, substrates are characterized by an N-terminal cleavable signal sequence. Protein folding takes place following signal peptide cleavage and translocation into the periplasmic compartment [13,16]. Numerous recombinant proteins have been successfully produced by utilizing the aforementioned system including Thermobifida fusca cutinase [17,18], phospholipase D [19], pullulanase [20] and archeal lipase [21]. Many factors such as protein stability, function, immunogenicity, and proper folding, etc. need to be considered in recombinant protein production. Unlike the cytoplasmic protein expression, extracellular protein secretion meets many of the above-mentioned requirements for the production of recombinant proteins. Protein secretion using type I and type II systems seems to be amenable to efficient and functional production of the rPF4, considering that it is small in size, bears two disulfide bonds, and is urgently needed to be produced in large scales. Therefore, a three-in-one system was designed enabling cytoplasmic protein expression or secretion via type I or type II systems through derivative constructs, allowing us to evaluate the potentiality of each system for large scale and functional production of the rPF4 protein.

## 2. Materials and methods

### 2.1 Strains and reagents

The restriction enzymes (XhoI, SalI, and NdeI) were obtained from Jena Bioscience (Jena, Germany), T4 DNA ligase was purchased from Thermo Fisher Scientific (Grand Island, NY). Anti-PF4 antibody (ThermoFisher Scientific Company, mouse monoclonal/IgG2b, clone name 170138, Grand Island, NY) and conjugated anti-mouse antibody (Abcam company, rabbit anti-mouse IgG, polyclonal, ab97046, USA) were used to perform the Western blotting experiments. Immobilized metal affinity chromatography (IMAC) using the Ni-NTA matrix (Qiagen) was employed for purification purposes. The synthetic construct was ordered from Biomatik Company (Biomatik, Ontario, Canada). To alleviate problems regarding *E*. *coli* codon bias, the Biomatik's proprietary codon optimization service was exploited. The agarose gel and plasmid extraction kits were purchased from Bioneer (South Korea). Isopropyl beta-D-thiogalactopyranoside (IPTG) and Kanamycin sulfate were acquired from Bio-Basic (Markham, Canada) and Merck (Germany) respectively. pET26b was utilized as the expression vector. BL21 (DE3) and DH5α strains of *E*. *coli* were provided by the Diagnostic Laboratory Sciences and Technology Research Center, Shiraz, Iran. The *E*. *coli* DH5α strain was employed for plasmid extraction purposes. The *E*. *coli* BL21 (DE3) strain was exploited as an expression host.

### 2.2 The DNA constructs

**Fig 1:**
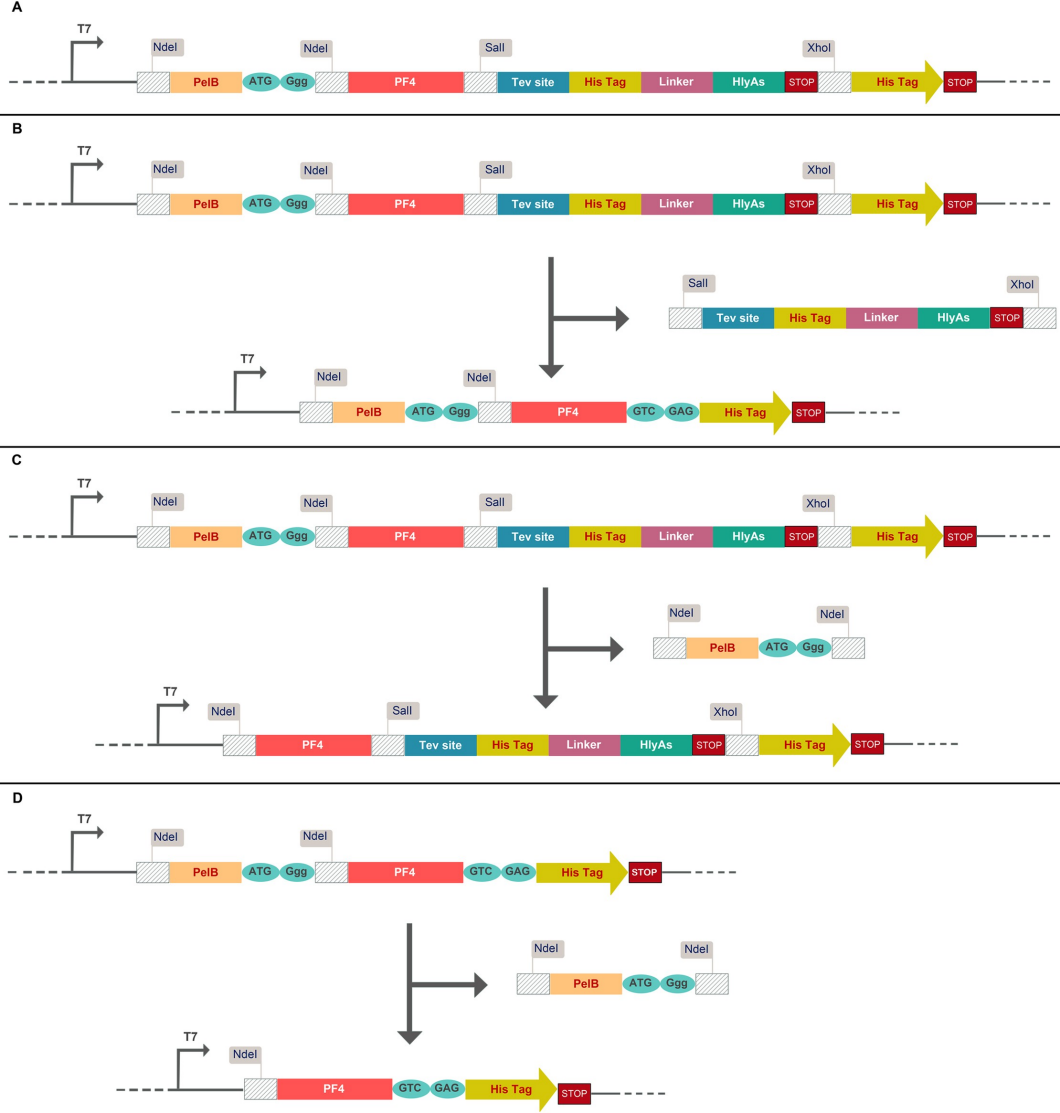
Schematic representation of the constructs employed in this study. (A) The underlying design principles of the three-in-one construct. The pelB and HLA signal sequences were placed upstream and downstream of the *PF4* coding sequence respectively. In addition, the *PF4* gene was fused to HlyAs through a linker sequence bearing a Tev recognition site. The restriction sites employed are indicated as flags. (B) Schematic representation of the T2SS construct. By sequential digestions using SalI and XhoI restriction enzymes the fragment harboring the HLAs and His-tag is removed, and the other His-tag along with the adjacent downstream stop codon (which are parts of the pET26b vector) are placed in frame with the *PF4* coding sequence, converting the primary three-in-one system into the T2SS construct. (C) Schematic representation of the T1SS construct. As the pET26b vector harbors an NdeI restriction site upstream of the pelB leader peptide, another NdeI site was placed at the 5ʹ end of the *PF4* coding sequence; as a result, through single digestion using NdeI, the fragment bearing the pelB sequence is excised out and the T1SS construct is generated. (D) Schematic representation of the construct enabling the cytoplasmic protein expression. The T2SS construct is subjected to NdeI digestion, resulting in a construct devoid of pelB and HLAs sequences.

The human *PF4* sequence (**accession Number: P02776**) and the *E*. *coli* hemolysin alpha (**accession Number: P08715**) signal sequence (HlyAs) corresponding to residues 965–1024 of α-hemolysin with the NdeI and XhoI restriction sites at the 5ʹ and 3ʹ termini and a SalI restriction site prior to HlyAs, was commercially custom-synthesized and cloned into pET26b at the NcoI and XhoI sites in the multiple cloning site of the vector. As part of the pET26b vector multiple cloning sites, a pelB signal sequence is placed upstream of the *PF4* coding sequence (Fig 1A). To obtain the constructs enabling protein export using type I or II secretion systems, the synthetic three-in-one construct was subjected to a series of digestions and ligations. The pET26b-pelB-rPF4 (pelB-rPF4) construct was made through sequential digestions using SalI and XhoI enzymes (Fig 1B) whereas the pET26b-rPF4-HlyAs (rPF4-HLAs) construct was obtained through single NdeI digestion (Fig 1C). Moreover, the pET26b-rPF4 (rPF4) construct was generated by digestion of the pET26b-pelB-rPF4 construct using the NdeI enzyme (Fig 1D). As the HlyA signal sequence needs to be free at its C-terminal end for proper functioning, the 6-His patch was included between the *PF4* gene and HlyAs through a linker sequence harboring a Tev recognition site. Located downstream of the HlyAs is a stop codon followed by another histidine patch and a second stop codon. After removal of the HlyA signal, the first stop codon is excised out, allowing the second histidine patch to be placed downstream of the *PF4* gene, followed by the second stop codon located in frame and enabling the expression of the pelB-rPF4-His-tag to halt. The ligation reactions were performed by T4 DNA ligase. All the reactions were carried out according to the manufacturers' protocol. Colony PCR assay, and nucleotide sequencing methods were employed to confirm the correct plasmid construction.

### 2.3 Investigating cytoplasmic expression of the rPF4 proteins mediated by the pelB-rPF4, rPF4-HLAs, and rPF4 constructs

Single colonies from the bacteria transformed with the previously prepared constructs were grown in 5 mL of Luria-Bertani broth (LB) containing 70 μg/mL Kanamycin separately, and incubated overnight at 37°C, 130 rpm. One hundred microliters of the starter cultures was transferred into 7 mL of fresh LB or TB (Terrific Broth) media containing 70 μg/mL Kanamycin. The cells were grown at 37°C, 150 rpm until reaching an optical density of 0.6–1.0 (OD 600). Then, protein expression was induced using 2 mM IPTG at 37°C, 200 rpm for 2 h. The cells were harvested by centrifugation at 5000 g for 15 min at room temperature.

### 2.4 Verification of protein expression using SDS-PAGE

The expression of recombinant proteins in the control and induced samples were analyzed using SDS-PAGE. The harvested bacterial cells were dissolved in denaturing lysis buffer (NaH2PO4, 100 mM, Tris-HCl 10 mM, Urea 8 M, and pH 8.0) and broken open using sonication. The resulting crude bacterial extract was centrifuged at 10000 g for 30 min at 4°C. Twenty microliters of the cleared supernatants corresponding to the rPF4, pelB-rPF4 or rPF4-HLAs constructs was mixed with 20 μl of Laemmli buffer (Tris-HCl 277 mM pH 6.8, Glycerol 44.4% (v/v), 4.4% SDS, 0.02% Bromophenol blue) and boiled at 100°C for 5 min. The samples were loaded onto a gel consisting of 13.5% (v/v) resolving and 5% (v/v) stacking parts. The samples were run at 80 V for 35 min to become stacked, and were further resolved at 150 V for 70 min. Finally, the gel was stained using Coomassie Brilliant Blue and the rPF4-HLAs (16 kDa), pelB-rPF4 (11.4 kDa; if the pelB sequence is retained and 8.8 kDa if the PelB signal is cleaved), and rPF4 (8.8 kDa) proteins were detected at the right locations. The actual molecular weights may differ slightly from the nominal weights.

### 2.5 Purification of the recombinant rPF4 protein

To further validate the rPF4 production, the proteins were purified employing the immobilized metal affinity chromatography method using Ni-NTA matrix. Cleared lysate was prepared as described in section 2.4. Lysis buffer contained 10 mM Imidazole and 0.5 M NaCl to decrease nonspecific protein binding due to the electrostatic interactions. The binding step was performed for 2 h with shaking at 200 rpm at the ambient temperature. The column was washed 2 times with the wash buffer (NaH2PO4, 100 mM, Tris-HCl 10 mM, Imidazole 10 mM, NaCl 0.5 M, Urea 8 M, pH 8.0) and four 0.5 mL eluates were collected using the elution buffer (NaH2PO4, 100 mM, Tris-HCl 10 mM, Imidazole 250 mM, NaCl 0.5 M, Urea 8 M, pH 8.0).

### 2.6 Recombinant rPF4 protein secretion mediated by type I or II bacterial secretory pathways

The secretion of the rPF4 mediated by type I or type II gram-negative bacterial secretory pathways was analyzed using a two set of seven mL *E*. *coli* BL21 cultures in fresh LB or TB media harboring either the pET26b-pelB-rPF4 or pET26b-rPF4-HLAs plasmids. The bacteria were grown according to section 2.3 and protein expression was triggered subsequently at a final concentration of 0.5mM IPTG at 29°C for 24 h.

### 2.7 SDS-PAGE analysis of the secreted proteins in the extracellular milieu of *E*. *coli* BL21 (DE3) cells harboring either the pelB-rPF4, rPF4-HLAs, or rPF4 constructs

To analyze type I- and II-mediated secretions, a three set of 10 mL *E*. *coli* BL21 cultures in fresh LB or TB media harboring either the pET26b-pelB-rPF4, pET26b-rPF4-HLAs, or pET26b-rPF4 were grown and protein expression was induced according to section 2.3 at a final concentration of 0.5 mM IPTG at 29°C, 200 rpm for up to 24 h. Subsequently, the TB and LB media expected to contain the secreted rPF4 protein were subjected to purification using immobilized metal affinity chromatography under native conditions. SDS-PAGE method was employed to analyze the secreted rPF4 protein purified from the extracellular milieu.

### 2.8 Verification of protein secretion using Western blotting

To further validate the rPF4 expression and secretion, we inspected the aqueous media for the presence of the recombinant proteins by Western blotting. Electrophoresis was performed on 13.5% gel according to section 2.4; however, in order to preserve at least a fraction of the higher structural states of the secreted rPF4 proteins (i.e. dimers, trimers, and tetramers) the boiling step was excluded from the sample preparation protocol. Western blotting was performed using specific anti-PF4 antibody. The protein bands were effectively blotted to the PVDF membrane using the semidry protocol carried out in Towbin transfer buffer (Tris base 24 mM, Glycine 190 mM, Methanol 20% (v/v)) at 20 V for 70 min. The membrane was blocked in 20mL blocking buffer (Tris 10 mM pH 7.4, NaCl 0.9% (w/v), 0.02% (v/v) Tween-20, 1% (w/v) skim milk) for 1 h on a rocking platform, and was further rinsed in 30 mL of Tris-buffered saline with tween-20 (TBST) (Tris 10 mM pH 7.4, NaCl 0.9% (w/v), 0.02% (v/v) Tween-20). Then, the membrane was immersed in anti-PF4 primary antibody diluted in blocking buffer (1:1000 dilution) for 60 min on a rocking platform at room temperature. The membrane was washed 6 times in TBST buffer. Next, the membrane was immersed in horseradish peroxidase-conjugated secondary antibody diluted in blocking buffer (1:1000 dilution) for 60 min and was subsequently rinsed by (6 times) TBST buffer. Finally, the protein bands of interest were visualized using either by tetramethylbenzidine (TMB) solution as the enzymes substrate or quantitative detections were carried out employing the enhanced chemiluminescence (ECL) method.

### 2.9 Investigating the protein secretion level as a function of IPTG, Triton X-100, glycine or sucrose supplementation

Four groups of 100-mL fresh TB cultures of *E*. *coli* BL21 (DE3) harboring the pET26b-pelB-rPF4 construct were grown according to section 2.3. After reaching an optical density (OD 600) of 0.6, each group of cultures, was divided into 10 mL flasks to individually analyze the effects of IPTG (ranging from 0.01–0.5 mM), glycine (ranging from 0–5% w/v), Triton X-100 (ranging from 0–3%w/v), and sucrose (ranging from 0–10% w/v). The cocktail media containing glycine, sucrose or Triton X-100 supplementations, were supplied with IPTG to a final concentration of 0.5 mM.

### 2.10 Investigating the trend of protein secretion over time

To investigate the optimum duration for secretion and to observe the trend of protein secretion over time, the protein content of the extracellular milieu was analyzed at several time points. To this end, 10 mL of induced culture of the bacteria harboring the pET26b-pelB-rPF4 construct, was subjected to analysis 5.5, 14, 20, 26, 39 and 48 h post-induction. In addition, to analyze the rPF4 stability in the extracellular environment and to further investigate the trend of the rPF4 protein secretion over time, 10-mL TB cultures of the bacteria containing either the pET26b-pelB-rPF4, pET26b-rPF4-HLAs, or pET26b-rPF4 plasmids and cultures of BL-21(DE3) devoid of any plasmid (NC) were grown according to section 2.3, and protein expression was induced by IPTG at a final concentration of 0.1 mM.

### 2.11 Investigating the synergistic effects of IPTG, Triton X-100, and glycine supplementation on protein secretion level

To further improve the secretion process, a fractional factorial experiment was employed to evaluate the combined effects of various concentrations of Triton X-100, glycine, and IPTG. To this end, a 400 mL TB culture of the bacteria containing the pET26b-pelB-rPF4 was prepared according section 2.3, and was further divided into 5-mL fractions in 36 flasks. Next, a combination of varied volumes of 100 mM IPTG, 10% glycine, and 10% Triton X-100 was added to the flasks until reaching the final concentrations of IPTG (ranging from 0.01–0.2 mM), glycine (ranging from 0–1% w/v), and Triton X-100 (ranging from 0–0.5% w/v), respectively. The flasks were supplied with an appropriate amount of the TB medium to a final volume of 10 mL and the flasks were further cultivated at 29°C with shaking (150 rpm) for 48 h.

### 2.12 The quantification of protein secretion using Image J software

The amount of the secreted rPF4 proteins were analyzed by Western blotting with the ECL detection method. The density of protein bands was determined by the Image J software. In order to figure out the concentration of protein bands from their respective density, serially diluted rPF4 with known concentrations ranging from 100 to 600 μg/mL was used. Next, the density was plotted against the concentration of proteins providing a standard curve which allows to quantitatively determine the amount of the secreted rPF4 proteins. As a measure of how well the regression predicts real data points, the plot’s coefficient of determination was calculated and found to be 0.98.

### 2.13 Analyzing the higher structural states of the secreted rPF4 proteins

In order to evaluate the capability of the secreted rPF4 protein to organize into its higher structural states, the SDS-PAGE and Western blotting methods were used. To this end, 20-mL TB cultures of the bacteria containing the pET26b-pelB-rPF4 were divided into 2 flasks with Triton X-100 concentrations of 0.1% and 0.5%. In addition, 10-mL TB cultures of the bacteria harboring the pET26b-rPF4 and 10-mL TB cultures of *E*. *coli* BL-21(DE3) (NC) devoid of any plasmids were grown according to section 2.3. The protein secretion was triggered at a final concentration of 0.1 mM IPTG for 48 h.

### 2.14 Investigating rPF4-heparin ultra-large complex formation and zeta potential measurement

The secreted rPF4 tetramerization and rPF4-heparin complex formation were studied using dynamic light scattering (DLS) technique (Nanopartica SZ 100; HORIBA Ltd, Kyoto, Japan) with a fixed 173 scattering angle and a 633-nm helium-neon laser. Data were analyzed using a Horiba SZ 100 apparatus for Windows [Z Type] software version 2.20 (Nanopartica SZ 100; HORIBA Ltd, Kyoto, Japan). Furthermore, Zeta potential experiments were performed, employing Horiba SZ 100.

### 2.15 Molecular modelling and structural alignment of the rPF4

Crystal structure of the PF4 protein (PDB ID: 4R9W) [22] was retrieved from the Protein Data Bank (PDB). Next, the three-dimensional structure of the rPF4 was constructed on the basis of the PF4 crystal structure as template and the primary structure of the rPF4 with a C-terminal His-tag. Homology modeling was performed using MODELLER 9.20 software [23] and one of the models generated was selected for subsequent studies based on DOPE scores. The resulting model was superimposed on the native PF4 structure and root mean square deviation (RMSD) values were calculated using UCSF Chimera 1.14 [24]. Furthermore, the pattern of hydrogen bonding with Fondaparinux (a structural analog of heparin) was also compared to the native structure with respect to a couple of key lysine residues [22] involved in attachment to heparin. The visualization of all models was carried out in UCSF Chimera 1.14 software.

### 2.16 Structural analysis of the secreted rPF4 proteins by Raman spectroscopy

The secreted rPF4 was subjected to lyophilization and the resulting powder was utilized in structural investigations. The experiment was performed at room temperature with a relative humidity of 20% using a LABRAM HR Raman spectrometer (Horiba Jobin Yvon, Villeneuve d’Ascq, France) coupled to a confocal microscope. The laser light at 532 nm (INNOVA 70C Series Ion Laser, Coherent, Santa Clara, CA) was used to illuminate the samples, and recording was carried out in 20 sec intervals. 100X objective lens was used to focus the laser beam on the lyophilized protein samples (0.9 NA, Olympus, Melville, NY). Spectral analysis was implemented using GRAMS/AI^™^ software (GRAMS SUITE 9.2, Thermo Fisher Scientific Inc.) and a linear baseline subtraction was performed in the amide I region spanning the 1600–1700 cm^-1^ range.

## 3. Results

### 3.1 The construction of rPF4, pelB-rPF4, and rPF4-HLAs

The recombinant pelB-rPF4, rPF4-HLAs (312 bp and 471 bp respectively) (Fig 1B and 1C) and the rPF4 (264 bp) (Fig 1D) constructs, were derived from the three-in-one construct, following digestion by NdeI, SalI, and XhoI as described in section 2.2. The correct construction of the vectors was ultimately confirmed by colony PCR and sanger sequencing.

### 3.2 The verification of the cytoplasmic rPF4 expression mediated by the rPF4, pelB-rPF4, and rPF4-HLAs constructs

**Fig 2:**
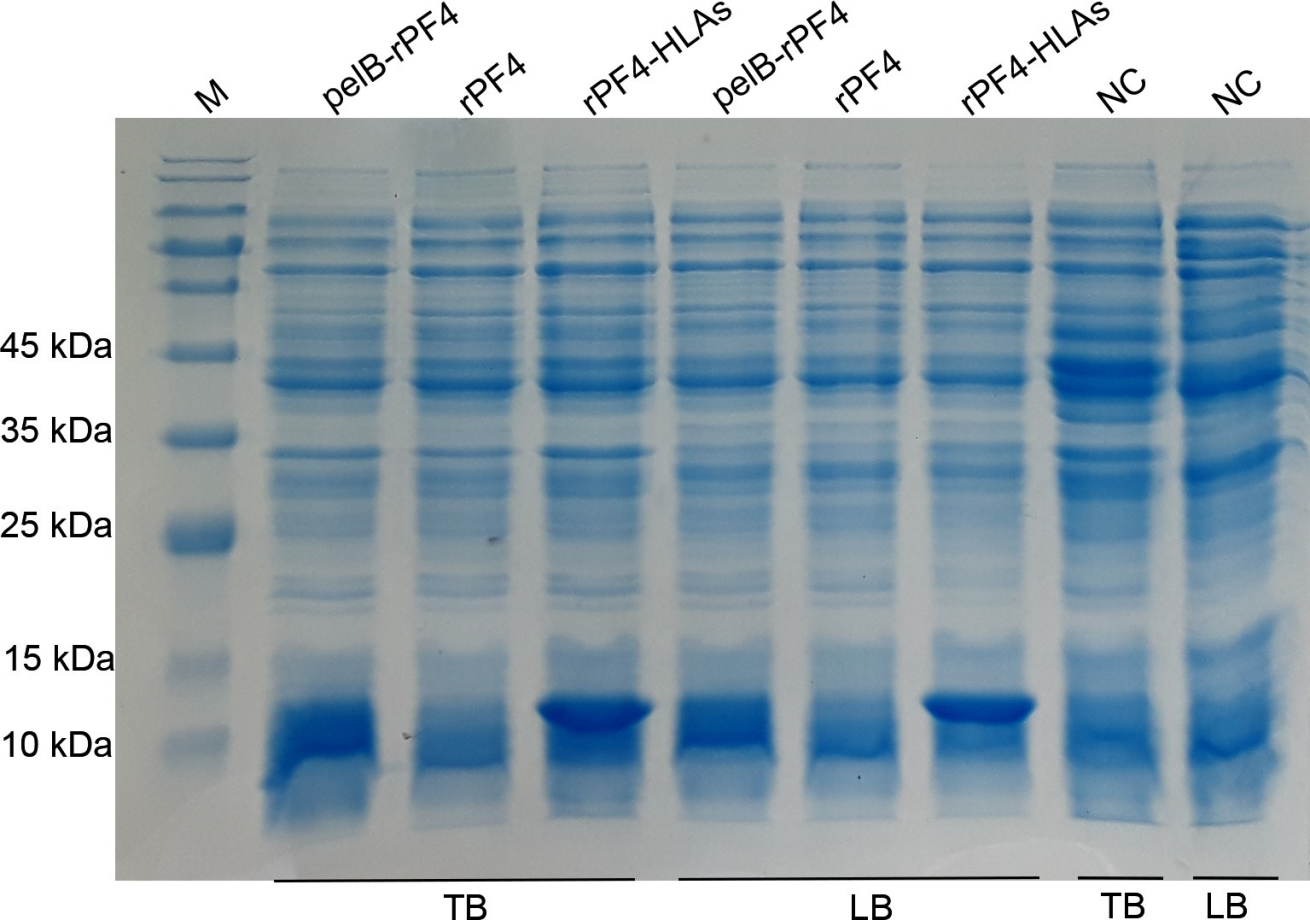
Analyzing the cytoplasmic expression of the pelB-rPF4, rPF4-HLAs, and rPF4 constructs. Expression was induced by 2 mM IPTG for 2 h in LB or TB media and the bacterial lysates were subjected to analysis using SDS-PAGE. All the constructs indicated an efficient cytoplasmic protein production; however, expression in the TB medium was found to be significantly improved compared to the LB medium. As an intrinsic property of the lac operon, a minor leaky expression from the rPF4 construct was observed in the non-induced groups. NC represents the non-induced groups serving as negative control. M denotes the molecular weight marker.

**Fig 3:**
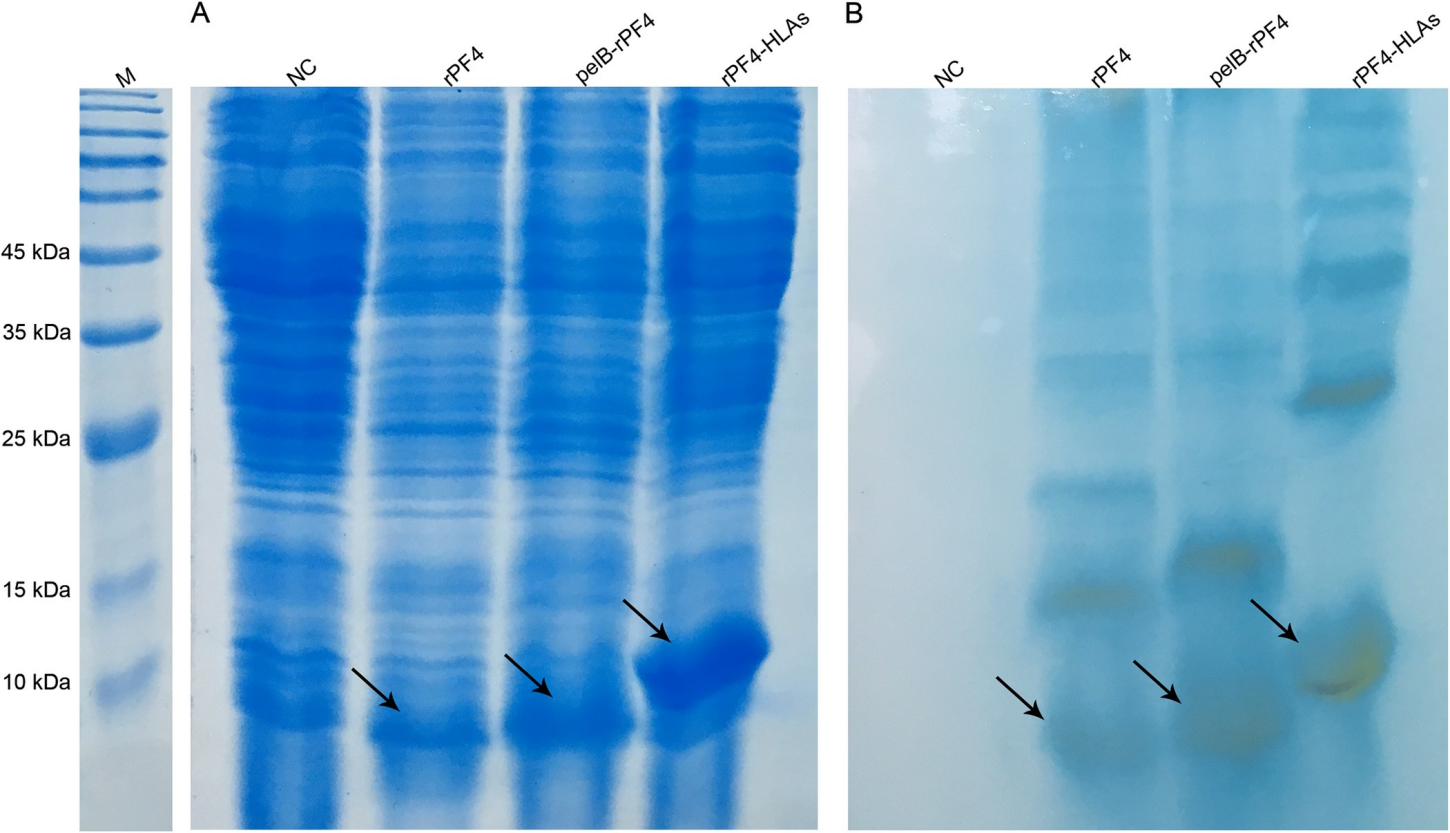
Western blotting confirmation of the cytoplasmic expression by the pelB-rPF4, rPF4-HLAs, and rPF4 constructs. The protein expression and sample preparation were carried out similar to the Fig 2. (A) Depicts the SDS-PAGE gel and (B) depicts the result of the Western blotting analysis performed on the same gel indicated in (A). Other bands visualized in the Western blotting analysis indicate various oligomeric states of the rPF4. In addition, compared to the other rPF4 variants, the migration of rPF4-HLAs protein has been retarded due to its additional HLA signal sequence. The rPF4 proteins respective to each construct was verified by specific anti-PF4 antibody. NC represents the negative control lacking the *rPF4* coding sequence. M denotes the molecular weight marker.

Protein expression was triggered by the addition of IPTG at a final concentration of 2 mM as described in section 2.3 and the bacterial lysate was subjected to SDS-PAGE analysis. Protein expression triggered at high concentrations of IPTG results in protein aggregation and inclusion body formation which is not amenable to SDS-PAGE analysis. This conundrum was resolved by taking advantage of 8 M urea to disentangle the inclusion bodies. Following sonication, the protein bands of interest corresponding to the rPF4, pelB-rPF4, and rPF4-HLAs with respective molecular weights of 8.8, 11.4 (8.8 kDa if the PelB signal is cleaved), and 16.2 kDa were detected in the soluble phase of bacterial lysate (Fig 2). Finally, in order to obtain a better resolution and to further corroborate the SDS-PAGE results of the rPF4 protein expression respective to each construct, Western blotting analysis was carried out (Fig 3).

### 3.3 The purification of the rPF4 protein expressed from the pET26b-rPF4 construct

**Fig 4:**
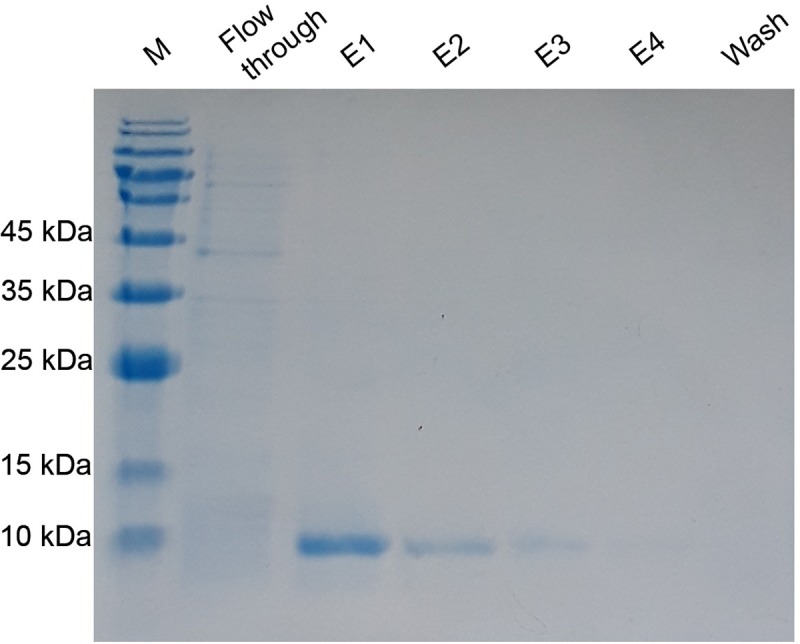
The purification of the rPF4 protein produced by the cytoplasmic expression system. E1 through E4 correspond to the cytoplasmic rPF4 proteins purified in 0.5 mL elution fractions. To avoid contamination with unwanted proteins, the matrix was washed three times with the wash buffer containing 10 mM of imidazole. M denotes the molecular weight marker.

To confirm the cytoplasmic expression of the rPF4 denaturing protein purification method was used. As revealed by the SDS-PAGE analysis on a 13.5% gel, the first elution harbored the bulk of the desired proteins with approximately 8.8 kDa in weight as outlined in Fig 4.

### 3.4 Verification of the rPF4 secretion mediated by pelB-rPF4 and rPF4-HLAs constructs by SDS-PAGE and Western blotting methods

**Fig 5:**
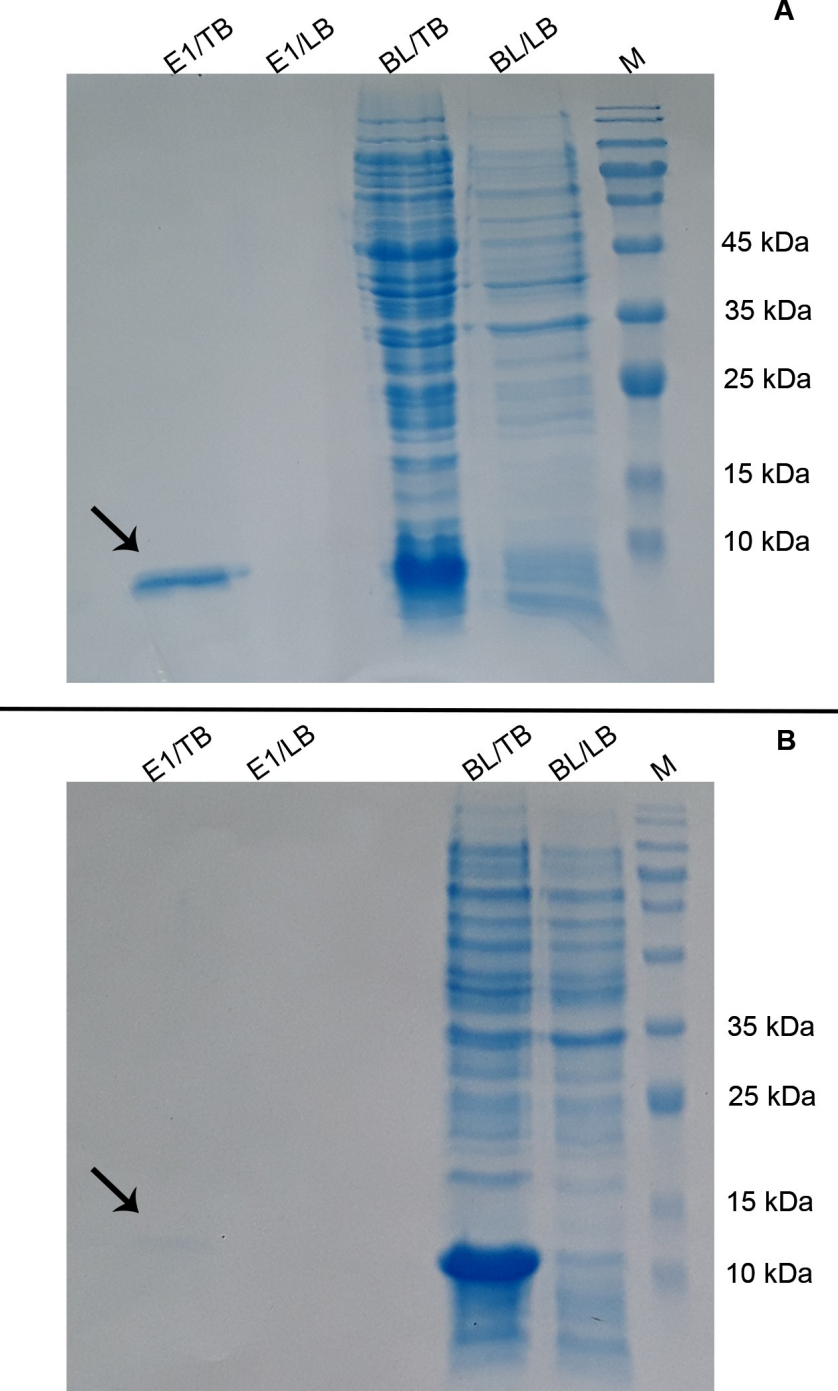
Investigating the rPF4 secretion through the T1SS and T2SS. Protein expression and secretion were carried out in 7 mL of LB or TB media for 18 h. Induction was triggered at 0.5 mM IPTG. BL refers to the cleared bacterial lysate. E1/TB and E1/LB indicate the secreted rPF4 proteins in TB or LB media respectively following purification. (A) Represents the T2SS-mediated rPF4 protein secretion into the extracellular milieu using pelB leader peptide. (B) Represents the T1SS-mediated rPF4 protein secretion into the extracellular milieu using HLA signal peptide. As demonstrated in the figures, the T2SS is more efficient in secreting the rPF4 compared to the T1SS. Furthermore, as confirmed by the SDS-PAGE analysis, growth in the TB medium enhances the protein secretion process and is superior to the LB medium.

**Fig 6:**
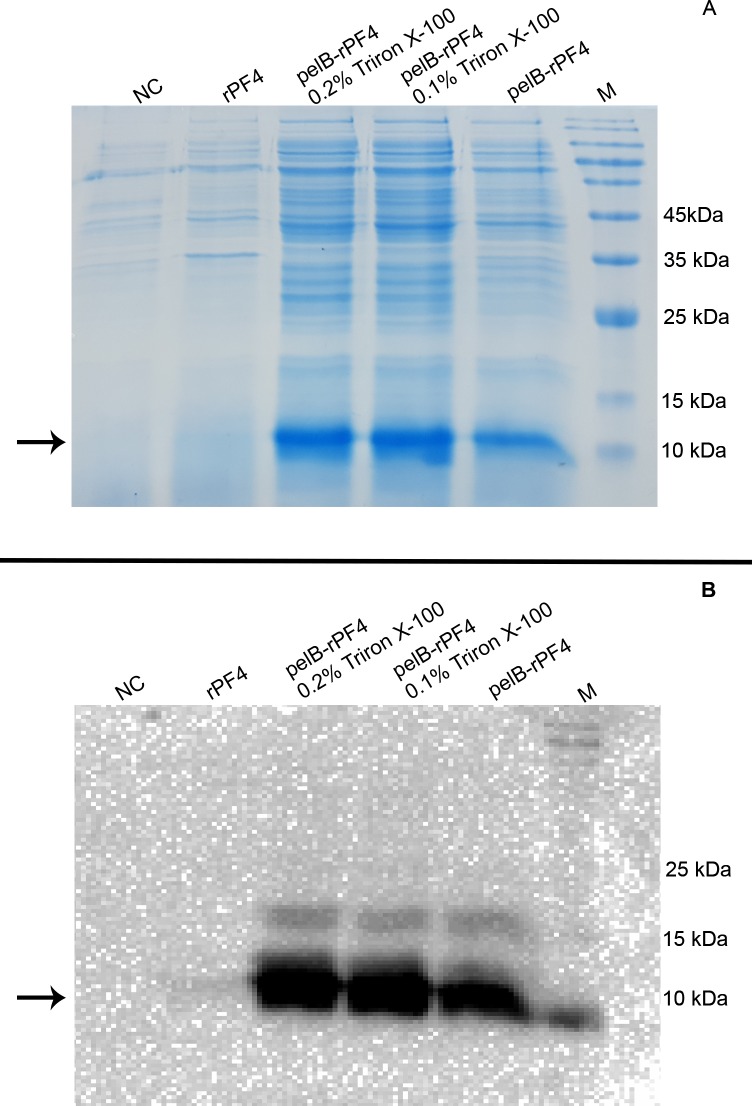
Confirmation of the rPF4 secretion into the outside medium using Western blotting. The rPF4 protein secreted into the extracellular milieu via the T2SS was subjected to SDS-PAGE (A) and Western blotting (B). Triton X-100 supplementation at low concentrations was found to increase protein secretion compared to the cultures lacking any Triton. The slight background regarding the pET26b-rPF4 construct is likely due to the cellular rupture during the 48-h cultivation or sample preparation. NC represents the negative control which lacks the *rPF4* coding sequence. The arrow indicates the rPF4 protein band. M denotes the molecular weight marker.

Induction at low IPTG concentrations and bacterial growth at low temperatures, would improve the protein folding process and obviate inclusion body formation which is important to be considered in protein secretion protocols. The bacterial cells were pelleted and the supernatant was separated. Subsequently, protein purification on TB or LB aqueous media was performed and the protein secretion into the extracellular milieu was analyzed by SDS-PAGE (Fig 5) The hosts bearing the pET26b-rPF4 construct were not able to secrete any protein into the extracellular environment. As depicted in Fig 5, the T2SS was more efficient than the T1SS in secreting the rPF4 protein into the extracellular milieu, and as a result, the T2SS was selected for producing the rPF4. Furthermore, as demonstrated by SDS-PAGE, protein expression and secretion were greatly enhanced in TB compared to the LB medium. Ensuing the SDS-PAGE analysis, the proteins were blotted onto the PVDF membrane and the rPF4 secretion was confirmed by Western blotting (Fig 6). In addition, Western blotting confirmed that low levels of Triton X-100 enhance protein secretion possibly through mild permeabilization of the outer membrane.

### 3.5 Individual effects of IPTG, Triton X-100, glycine, and sucrose on protein secretion rate

**Fig 7:**
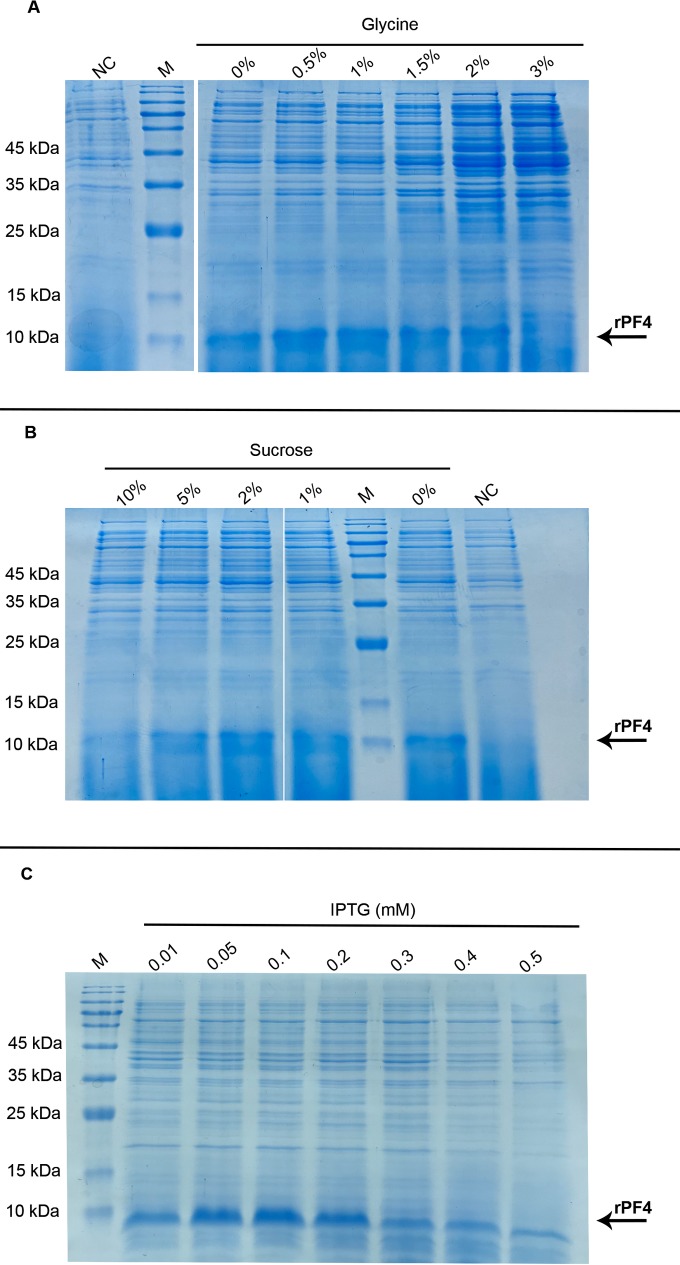
The individual effect of various chemical supplements on protein secretion. (A) Represents the effect of glycine supplementation on protein secretion. Glycine supplementation mildly improved the release of the rPF4 into the extracellular space. Secretion at 0.5% glycine concentration demonstrated the highest level. (B) Represents the effect of sucrose on protein secretion. In contrast to glycine, sucrose supplementation negatively affected the secretion of the rPF4. (C) Fine tuning the IPTG concentration used to induce protein secretion. IPTG was used at concentrations from 0.01–0.5 mM, and 0.1 mM IPTG was found to be the optimum concentration to result in the best secretion level.

Initially the effect of various amounts of glycine on protein secretion (ranging from 0–5% w/v), was analyzed. The level of protein secretion was mildly augmented with 0.5% glycine supplementation (Fig 7A) Further supplementation with 3%, 4%, and 5% glycine inhibited culture cell Growth. Furthermore, supplementation with 5% or 10% sucrose negatively affected the rPF4 release into extracellular milieu, and lower concentrations did not reflect a notable change in protein secretion (Fig 7B). Moreover, supplementation with Triton X-100 at higher concentrations (1%, 2% and 3%). was also found to completely inhibit growth. Finally, the effects on protein secretion of IPTG at concentrations ranging from 0.01–0.5 mM was explored. The addition of IPTG at concentrations ranging from 0.01–0.2 mM was found to enhance protein secretion, with higher concentrations negatively affecting the protein secretion rate (Fig 7C).

### 3.6 The trend of protein secretion over time

**Fig 8:**
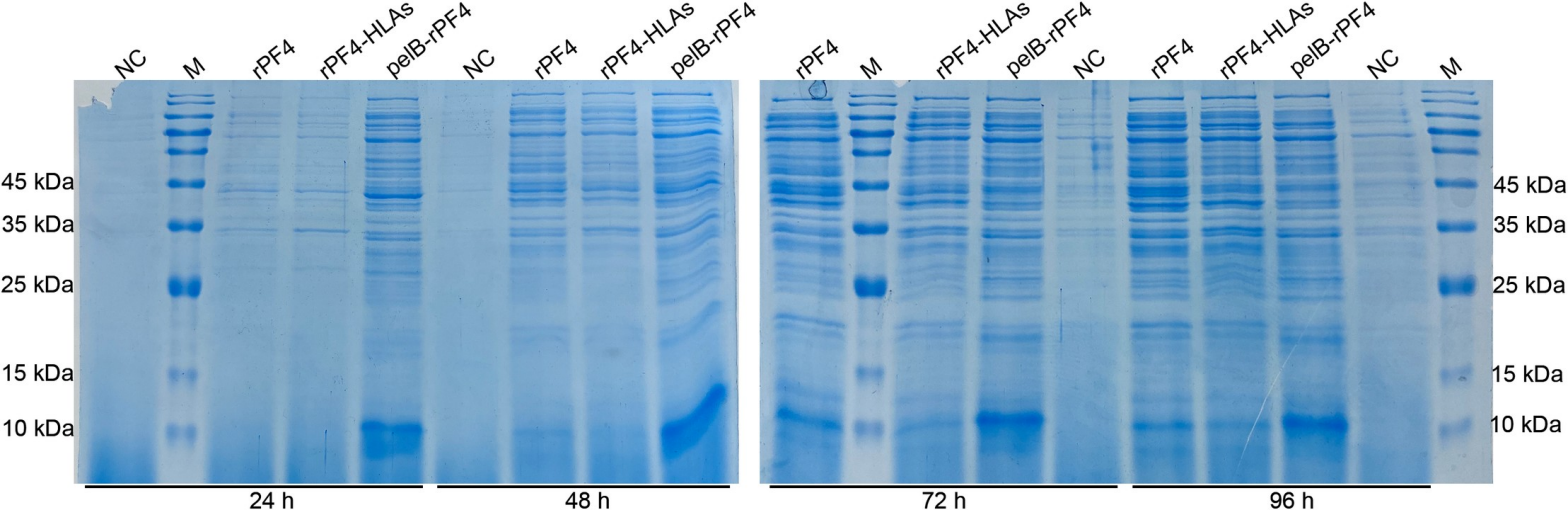
Investigating the stability of the secreted rPF4 as a function of time. The lanes labeled by rPF4, pelB-rPF4 and rPF4-HLAs refer to proteins expressed from the pET26b-rPF4, pET26b-pelB-rPF4 and pET26b-rPF4-HLAs constructs, respectively. Bacteria harboring the pelB-rPF4 were found to secrete the highest amount of rPF4. The secretion seems to be stable until 48 h, and no considerable increase in the rPF4 secretion was observed beyond 48 h. The background secretion from the pET26b-rPF4 construct started to increase after 48 h and seems to be due to the cellular rupture as a result of excessive cultivation or sample preparations (for instance, the centrifugation step). NC indicates the negative control and the M denotes the molecular weight marker.

The protein secretion trend in bacterial cells harboring the pelB-rPF4 construct was analyzed as a function of bacterial density and was observed to increase over time during the first 20 h but decline thereafter, indicating that the increase in protein secretion rate is considerably slower than the bacterial growth (S1 Fig). Furthermore, the rPF4 stability was found to be optimal until 48 h post-induction. However, with secretion beyond 48 h, a notable presence of nonspecific proteins other than the target rPF4 were observed, specifying 48 h as a more optimal secretion duration (Fig 8).

### 3.7 The effect of culture scale up on T2SS-mediated protein secretion and purification of the rPF4

**Fig 9:**
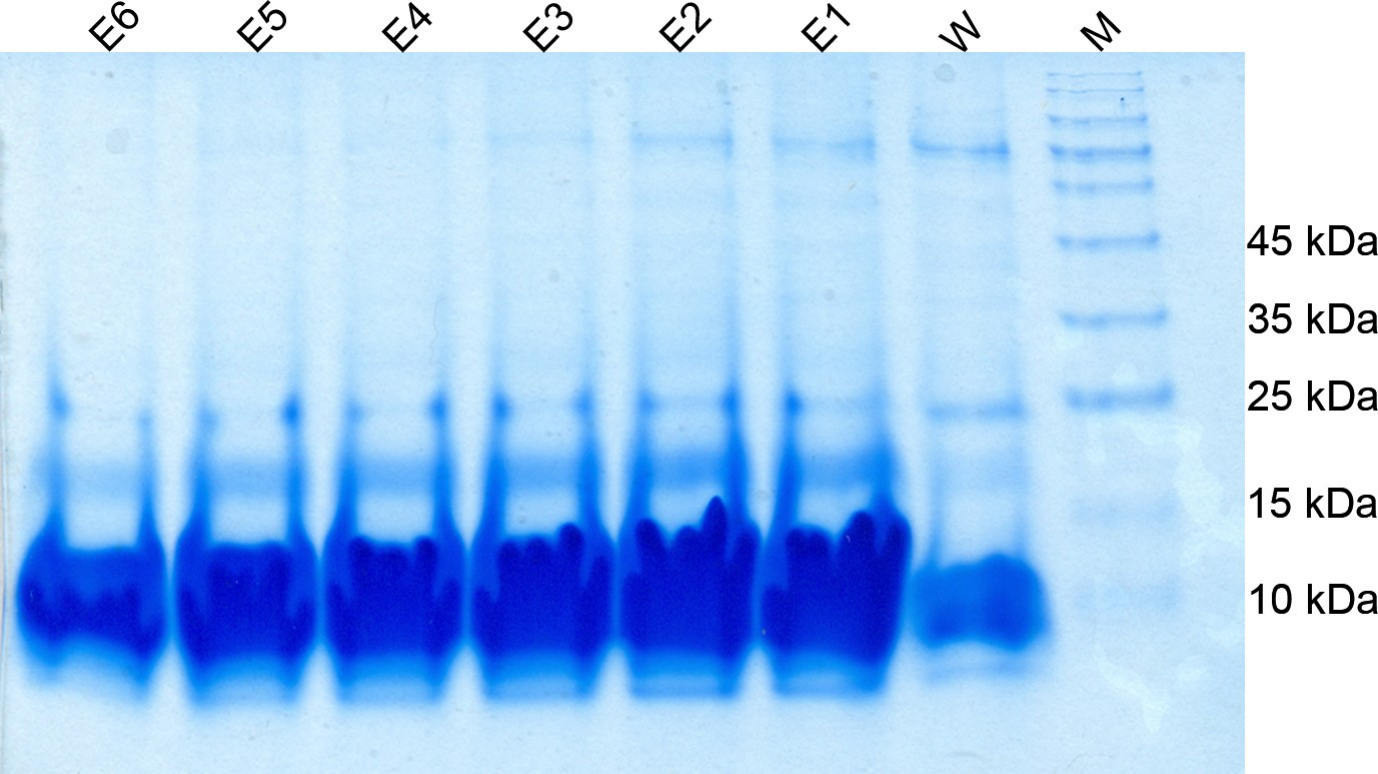
The SDS-PAGE analysis of protein secretion following purification using the Ni-NTA system. The bacteria harboring the T2SS construct were grown in 40 mL of the TB medium, induced and their supernatant was subjected to purification. Protein secretion was triggered at 0.1 mM IPTG and continued for 48 h. The purification column was washed with 10 mL of the wash buffer containing 10 mM Imidazole. Then, proteins were eluted in 1 mL fractions, designated as E1 through E6.

Considering that cost-effectiveness along with achieving the highest yield are important criteria to meet when dealing with recombinant protein production, the bacterial culture was scaled up to 40 mL in the absence of supplementation. As mentioned previously, induction was performed at low IPTG concentration (0.1 mM). The secretion process continued for 48 h and the aqueous media was subjected to protein purification. Proteins were eluted in 2mL fractions, and over 12 mg of the rPF4 was purified from the media under native conditions (Fig 9).

### 3.8 The synergistic effects of IPTG, glycine and Triton X-100 on the trend of protein secretion

**Fig 10:**
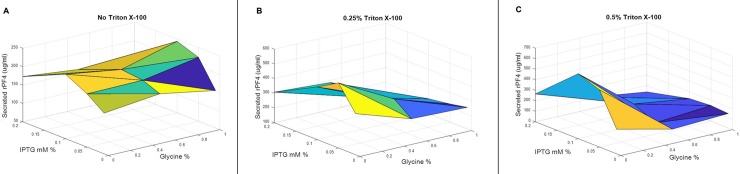
A three-dimensional representation of the synergistic effects on the rPF4 secretion of supplementation with various concentrations of glycine, Triton X-100, and IPTG. (A) In the absence of Triton X-100, the best secretion level is achieved by moving toward lower IPTG concentrations near 0.1 mM. In addition, tweaking glycine concentration does not seem to result in any significant change in the protein secretion level. (B) When the cocktail medium is supplemented with 0.25% Triton X-100, the optimum secretion point is achieved by moving toward zero glycine concentration and 0.1 mM IPTG. (C) At 0.5% Triton X-100 concentration, the optimum secretion level is brought about at zero glycine concentration and 0.05–0.1 mM IPTG.

**Fig 11:**
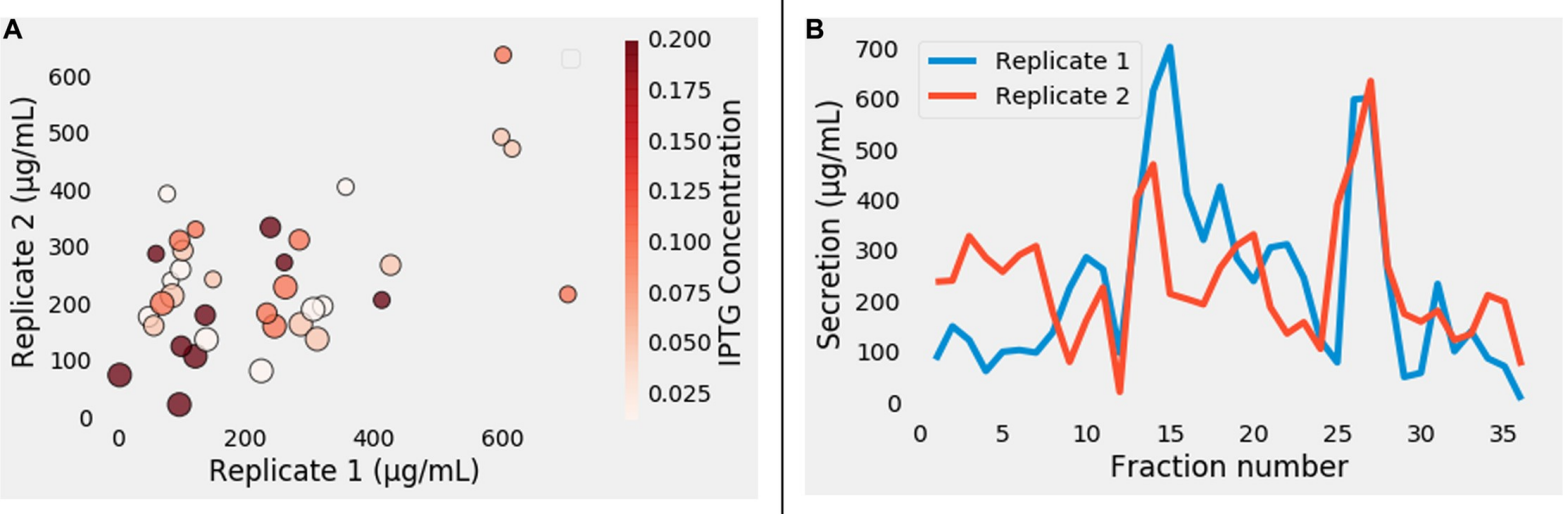
Evaluation of the secretion trend and reproducibility. (A) Indicates the trend of protein secretion in various fractions. (B) To evaluate the reproducibility of secretion replicates, Pearson correlation between the two sets was calculated and the secretion experiments were found to be in good agreement as revealed by a Pearson correlation coefficient of 0.5 and p-value of 0.001.

**Fig 12:**
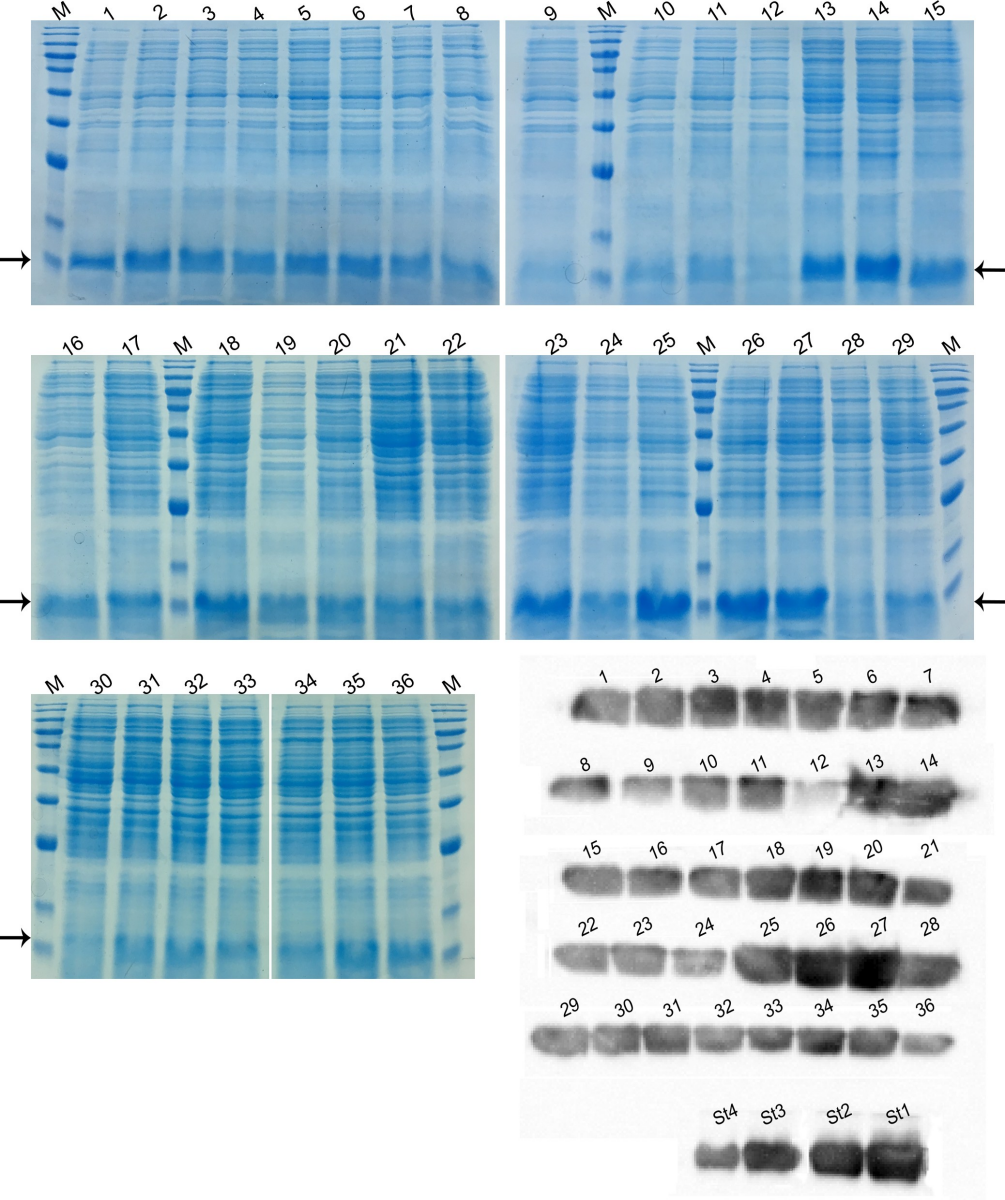
SDS-PAGE and Western blotting analysis of the synergistic effects of glycine, Triton X-100, and IPTG. The various fractions used in the factorial analysis were subjected to SDS-PAGE and Western blotting analysis. The fractions are labeled 1 through 36. A known concentration of the rPF4 was serially diluted and served as standard in order to extrapolate the amount of secretion in each fraction. Serially diluted rPF4 bands are labeled St1 through St4 in order of decreasing concentration. The rPF4 bands are indicated by arrows, M denotes the molecular weight marker.

A fractional factorial experiment was devised to examine the synergistic effects of supplementation with IPTG, glycine, and Triton X-100 on the level of the rPF4 secretion (Table 1). To facilitate the experiment, sucrose was excluded from the analysis as the primary results indicated its adverse effects. Since the secretion level was negatively influenced by higher concentrations of IPTG, the fractions induced at 0.3, 0.4 and 0.5 mM of IPTG were eliminated from the experiment. Furthermore, to reduce the detrimental effect of Triton X-100, the effect of lower concentrations was investigated. Protein secretion in media triggered at 0.1 mM IPTG and supplemented with 1% glycine approximately yielded 244 μg/mL of the rPF4. Protein secretion triggered at 0.05 mM IPTG with 0.25% Triton X-100 supplementation approximately resulted in 542 μg/mL of the rPF4. Additionally, protein secretion triggered at 0.1 mM IPTG with 0.5% Triton X-100 supplementation approximately resulted in 617 μg/mL of the rPF4 (Figs 10, 11 and 12).

**Table 1:** The amount of the rPF4 protein secreted in the fractional factorial experiment (μg/mL).

Triton X-100%	Glycine %	IPTG (mM)							
		n	0.01	n	0.05	n	0.1	n	0.2
0	0	1	160.73	2	194.71	3	225.07	4	172.95
	0.5	5	178.21	6	196.66	7	202.90	8	157.32
	1	9	151.98	10	223.63	11	244.29	12	58.60
0.25	0	13	379.24	14	542.23	15	457.71	16	307.97
	0.5	17	256.61	18	345.52	19	295.60	20	285.09
	1	21	246.29	22	223.43	23	201.44	24	113.52
0.5	0	25	234.31	26	543.68	27	617.66	28	265.07
	0.5	29	111.82	30	108.11	31	206.86	32	111.26
	1	33	136.92	34	148.68	35	134.21	36	38.14

n represents the fraction number assigned to each condition. The data presented in the table is the mean value of the two independent experiments.

### 3.9 Investigating the higher structural states of the secreted rPF4 proteins

**Fig 13:**
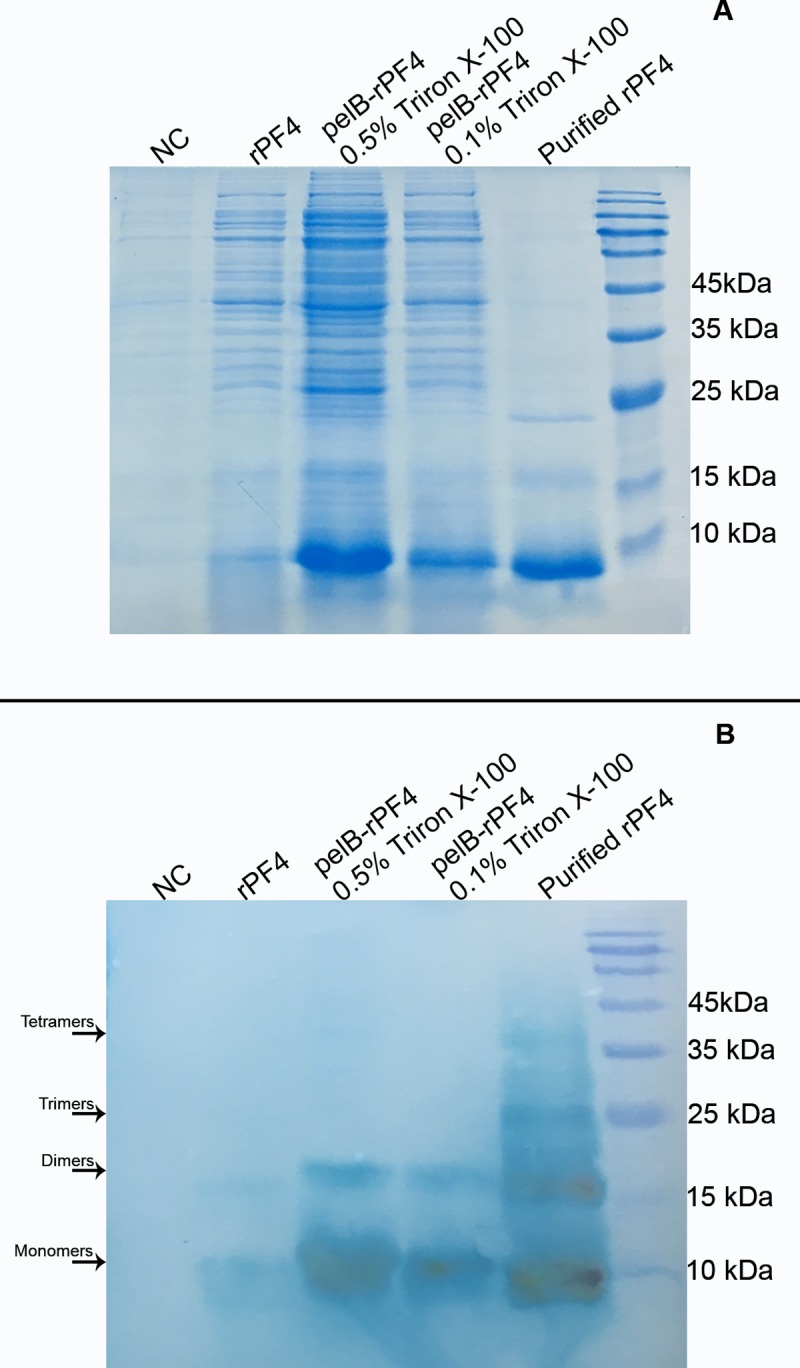
Investigating the formation of higher structural levels by the secreted rPF4. The lanes labeled as rPF4 and pelB-rPF4 represent proteins expressed from the pET26b-rPF4 and pET26b-pelB-rPF4 constructs respectively. The purified rPF4 devoid of any detergents delineates all the oligomeric forms of rPF4; however, in samples containing 0.1% and 0.5% Triton X-100, the monomeric and dimeric states constitute the sole structural states of the secreted proteins. NC represents the negative control and the M denotes the molecular weight marker.

Different structural organizations of the secreted rPF4 was confirmed via the Western blotting method. The purified secreted rPF4 proteins were found to possess the entire set of oligomeric states of the protein; however, samples supplemented with various concentrations of Triton X-100 solely indicated the monomeric and dimeric states of the rPF4, indicating that the presence of Triton disrupts the trimeric and tetrameric forms of the rPF4 which are less stable than the dimeric and monomeric forms. The results also revealed that due to the exclusion of the boiling step from the sample preparation protocol, a portion of the secreted rPF4 had retained its higher structural levels including dimers, trimers, and tetramers. (Fig 13). However, if the purification procedure is performed in buffers lacking Triton X-100, the secreted proteins are able to organize to their quaternary structure again.

### 3.10 The rPF4 tetramerization, rPF4-heparin ultra large complex formation, and zeta potential analysis

The size distribution of the complexes formed between rPF4 and heparin was investigated by dynamic light scattering (DLS). DLS is governed by the Brownian motion of particles and is inversely related to the particles’ size. In the absence of heparin, the rPF4 at concentrations of 100, 200, and 400 μg/mL formed small particles of approximately 10 nm in size which is correlated to tetrameric oligomerization of the protein. Larger complexes of approximately 100 to 1200 nm in size began to form at 5 to 20 units of heparin in response to different concentrations of the rPF4 by incubation for 15 to 120 mins. Furthermore, overnight incubation of the rPF4 at 200 μg/mL concentration with 5 units of unfractionated heparin (UFH) produced larger complexes of roughly 600 and 1200 nm in size (Table 2) (S2, S3 and S4 Figs). Altogether, the results indicate that the concentrations of rPF4 and UFH, along with the temperature at which the mixtures are incubated are important factors affecting the ultra large complex formation. Zeta analysis of 600 μg/mL of the rPF4 confirmed a positive charge of approximately 98 mV (S5 Fig).

**Table 2:** The rPF4 tetramerization, heparin-rPF4 complex formation, and oligomerization.

rPF4 (μg/mL)	UFH (unit)	Temperature (°C)	Incubation Time (min)	Size (nm)	Standard deviation (SD)
100	-	25	120	8.7	± 0.3
100	-	25	120	14.5	± 0.8
100	20	25	120	132	± 8
100	20	25	120	86	± 3.4
200	-	25	Overnight	8.9	± 3.1
200	-	37	Overnight	12.8	± 5.2
200	5	25	Overnight	643	± 68.6
200	5	37	Overnight	1260	± 106.7
400	-	25	15	7.8	± 3.4
400	5	25	15	90	± 28.6
400	10	25	15	126	± 47

When purified rPF4 was subjected to DLS, the size of particles detected span a range of ~8–14 nm in size which indicates the formation of tetramers by the secreted rPF4. When the rPF4 was mixed with heparin, oligomeric structures ranging in size from ~90–1200 nm were detected indicating that the tetrameric rPF4 proteins acquired functional quaternary structures and were able to undergo oligomerization in the presence of the UHF. The size of the oligomeric structures is commensurate with an increase in incubation time and temperature up to 37°C.

### 3.11 Molecular modelling and structural alignment of the rPF4

**Fig 14:**
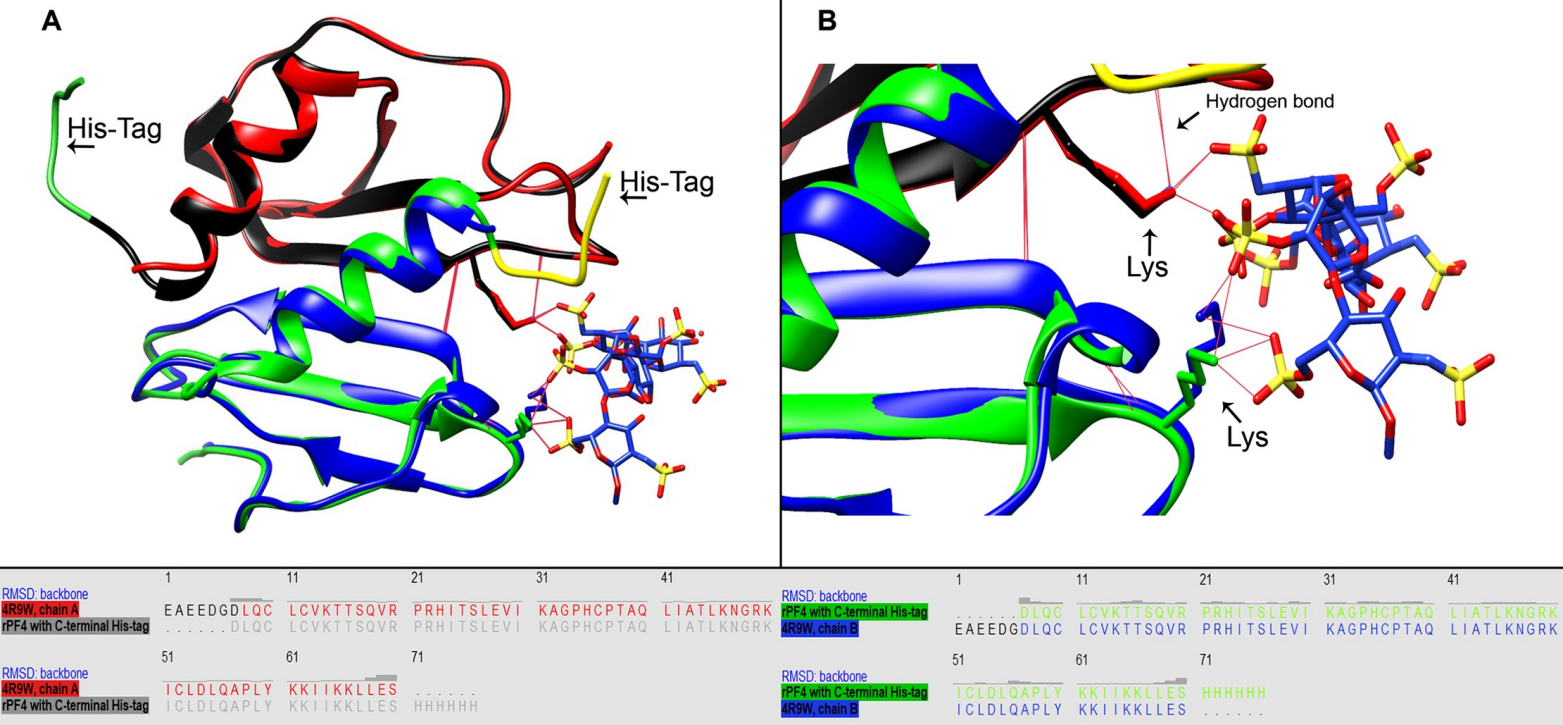
Molecular modelling and structural alignment of the rPF4. The rPF4 models are shown in black and green, with their corresponding C-terminal His-tags illustrated in green and yellow, respectively. Red bars indicate hydrogen bonds and the critical lysine residues are labeled with black arrows. The backbone RMSD values are illustrated above each amino acid residue.

MODELLER 9.20 software was used to perform the homology modelling experiments and the model with a DOPE value of -0.17 was selected. Structural superimposition of the rPF4 model (including a His-tag at its C-terminus) indicates a near perfect alignment of the native and recombinant PF4 proteins as revealed small backbone RMSD values. In addition, two lysine residues with critical roles in attachment to heparin were selected for further analysis and the pattern of hydrogen bonds were found to be in good agreement with that of native PF4 (Fig 14).

### 3. 12 Structural analysis of the rPF4 via Raman spectroscopy

**Fig 15:**
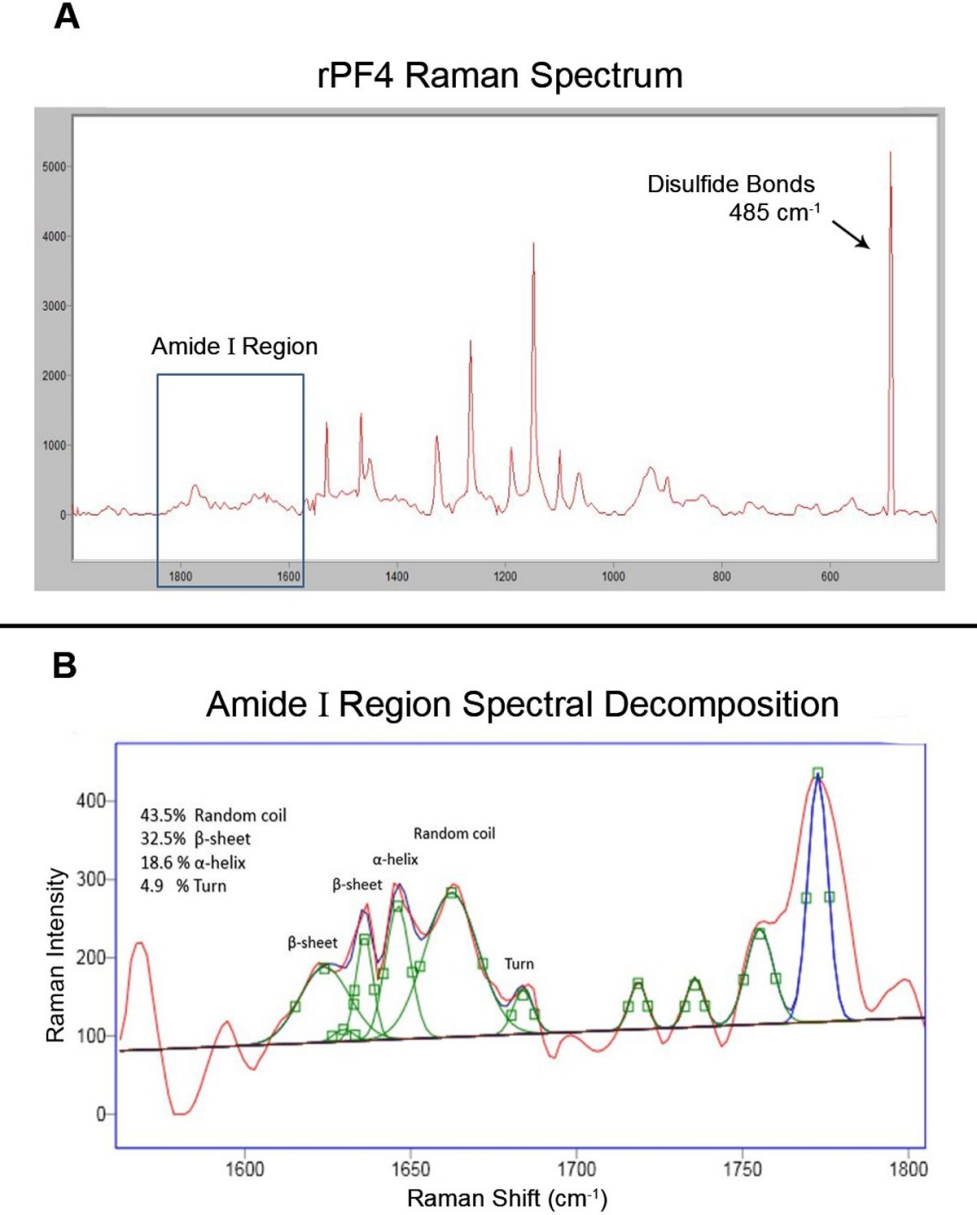
Investigation of the secreted rPF4 secondary structure composition and disulfide bond formation. By exploiting Raman spectroscopy, the conformation of the secreted rPF4 was analyzed. (A) Represents disulfide bond formation at the 485 cm^-1^ of Raman shift. (B) Following decomposition of the amide I region, the secondary structure composition of the rPF4 was revealed to be in near perfect agreement with predictions based on x-ray crystallography data.

To ascertain if the rPF4 proteins are secreted in their native conformation, 2 mg of the rPF4 was freeze dried and subjected to Raman spectroscopy. The spectrum was analyzed in different Raman shifts to identify disulfide bond formation and secondary structure composition of the rPF4. Following decomposition and linear base line subtraction in amid I region, the rPF4 was revealed to be approximately comprised of 43.5% Random coil, 32.5% β-sheet, 18.6% α-helix, and 4.9% turn, which is in concordance with the crystal structure of the PF4 deposited in the protein data bank. The native PF4 structure is comprised of two disulfide bonds with pivotal roles in the native functional conformation of the protein and by careful analysis of the Raman spectrum, the formation of these two critical disulfide bonds was confirmed (Fig 15).

## 4. Discussion

*E*. *coli* has been the chief bacterial host for the production of recombinant proteins with commercial and medical significance. A kaleidoscopic variety of genetically engineered hosts, expression vectors, and expression strategies have made this process significantly convenient [25,26]. Intracellular protein expression has been in widespread use, albeit complicated by hurdles including inclusion body formation [27], protein degradation due to protease release, and liberation of lipopolysaccharide (LPS), demanding cumbersome downstream procedures such as denaturation and refolding[28], endotoxin removal [29,30] and successive steps of protein purification [31].

Extracellular protein production seems to be promising as it has the potential to circumvent the need for breaking open the cells, to enhance protein purity, and to pave the way for the large-scale industrial protein production [13]. We envisioned that the T1SS and the T2SS secretory pathways of *E*. *coli* would be suitable for the production of recombinant PF4. Several factors contribute to the superiority of recombinant protein secretion in *E*. *coli* over the conventional cytoplasmic expression. Target proteins are sequestered away from a variety of destructive proteases through protection by the components of the secretory machinery. SecB, as a pivotal component of the type II secretory pathway, grabs onto and holds the target protein in a semi-folded conformation thus retaining their competency for the post-translational translocation through the inner membrane into the periplasmic space, thereby preventing protein aggregation [32]. Based on previous studies indicating the dependency of the pelB signal sequence on secB to direct protein secretion [18], we speculate that coexpression of the secB protein may also improve the secretion level of rPF4 via type II secretory pathway by augmenting the T2SS machinery through providing surplus of secB proteins, compensating for the rate-limiting amount of secB at disposal. Furthermore, based on our observations, in the case of toxic recombinant proteins, the secB protein sequesters the target proteins and diminishes their toxicity to a large extent (unpublished data). As opposed to the highly crowded intracellular area where the mean distance between the proteins is less than the size of an average protein which results in a huge thermodynamic driving force for protein aggregation [33,34], the ample extracellular space provides the exported recombinant proteins with enough room to accumulate and remain in their native state.

Although a considerable amount of rPF4 was observed to be secreted into the extracellular space, we envisioned that the periplasmic environment is still teeming with large quantities of rPF4, and to release the proteins trapped there while continuing to preserve the integrity of the outer membrane and minimizing the risk of LPS contamination, the expression media were supplemented with small amounts of glycine and Triton X-100. Triton X-100 supplementation was revealed to be highly effective presumably by slightly increasing the outer membrane permeability. Furthermore, as IPTG triggers protein expression through derepression of the T7 promoter, we sought to evaluate the optimum derepression allowing the components of the secretory apparatus to keep up with the pace of protein production so as to achieve maximum protein secretion. At higher concentrations of IPTG, the secretary system gets exhausted and fails to assist in efficient protein secretion, and as consequence a lower secretion yield is observed. On the contrary, a very mild induction of protein expression favors the protein folding process, solubility, and an efficient transport into the extracellular milieu.

A higher proportion of chaperones residing in the periplasmic environment [32] creates a more favorable compartment for protein folding and correct disulfide bond formation, particularly when dealing with sophisticated eukaryotic proteins with more complicated folding kinetics. Therefore, by exporting the recombinant proteins into the periplasm, there is a higher likelihood of the formation of native functional state by recombinant proteins. From a different perspective, it is notable that the lower levels of proteases in the periplasm boosts protein stability by avoiding unwanted degradation [13]. Furthermore, cleavage by the signal peptidase (for instance the Lep protease) of the N-terminal signal sequence leads to an authentic N-terminus by rendering the target protein devoid of the initial Methionine, restriction enzyme scar sites and other unwanted junk sequences that may adversely affect the native protein structure and function [35].

A variety of measures were taken to mitigate the shortcomings of cytoplasmic recombinant PF4 production to achieve enhanced solubility, higher yields, robust folding, efficient oligomerization, and increased purity, through the cumbersome manipulation of intracellular expression systems and successive steps of technically demanding protein purification strategies. In an effort to produce a recombinant form of PF4 with antigenic similarity to its native variant, several chemicals were employed to enhance protein solubility and yield[36]. In another study, an effort was made to augment the PF4 protein expression by placing its encoding sequence in several tandem repeats [37]. Although these methods seemed to remedy some of the shortcomings, producing the recombinant form of PF4 lacking its native disulfide bonds appears to be suboptimal. Furthermore, handling the large volume of cell culture or introducing tandem repeats of PF4 genes into the construct in order to increase the protein yield mainly gave rise to inclusion body formation. However, extracellular recombinant PF4 production was found to more effectively alleviate many of the aforementioned downsides compared to intracellular protein expression. Ultra-large complex formation between unfractionated heparin and secreted rPF4 revealed a high protein solubility, proper folding, and efficient formation of tetramers, as confirmed by dynamic light scattering, Raman spectroscopy, and Western blotting.The secretion of the recombinant PF4 to the extracellular medium also circumvents the arduous task of removing LPS as it preserves the bacterial cell integrity by avoiding the need for bacterial cell wall rupture. Proper disulfide bond formation has always been a major concern in cytoplasmic protein expression methods, which commonly demands difficult downstream procedures. However, by harnessing the bacterial secretory apparatus, these conundrums are overcome to a large extent.

Based on the quantity of protein mass accumulated in the bacterial culture, the classical HylA T1SS of *E*. *coli* directly ferrying recombinant rPF4 proteins to the extracellular milieu was found to be inferior to the SecB-dependent T2SS. The low expression levels and early exhaustion of the molecular constituents involved in the classical HylA T1SS might be the rate-limiting agents and thus accounted for the inefficiency of the rPF4 secretion using this system. It is envisaged that an all-in-one vector furnishing a surplus of these restrictive components might boost the system’s capacity for a more efficient T1SS-based export of the recombinant PF4 outside the cell.

In this study, the Ni-NTA purification system was employed but shifting to an alternative approach including heparin affinity chromatography circumvents the need to add a patch of extra Histidine amino acids, and therefore resulting in exact structural similarity with the native platelet-derived factor.

## 5. Conclusion

In conclusion, employing an extracellular protein production approach facilitates the PF4 production, purification, and biochemical analysis to a large extent which remedies the many shortcomings associated with conventional cytoplasmic expression of recombinant proteins and promises a more efficient and cost-effective approach for future industrial applications.

## Supporting information

S1 Fig The trend of protein secretion over time. Protein secretion as a function of bacterial density. Bacterial density follows an increasing trend for the first 20 h, and begins to decline then after, indicating that the total protein secretion has a slower pace than the bacterial growth. The vertical axes, indicates secretion per bacterial density, in μg mL^-1^ OD^-1^.
(DOCX)

S2 Fig Oligomerization analysis at 100 μg/mL concentration of rPF4. 100 μg/mL of rPF4 was subjected to DLS measurements. Only C and D conditions were supplemented with 20 units/mL of UFH. All the conditions were incubated for 2 h at 25°C. Approximately 100 nm of large complexes formations were induced between UFH and rPF4 tetramers.
(DOCX)

S3 Fig Oligomerization analysis at 200 μg/mL concentration of rPF4. 200 μg/mL of rPF4 was subjected to DLS measurements. Only C and D conditions were supplemented with 5 units/mL of UFH. All the conditions were incubated overnight. Conditions A and C were stored at 25°C, whereas conditions B and D were stored at 37°C. Very large complexes of 600 nm to 1200nm were formed between UFH and rPF4 tetramers. Storing at 37°C appears to induce a larger complex formation than 25°C.
(DOCX)

S4 Fig 400 μg/mL concentration of rPF4 oligomerization analysis. 400 μg/mL of rPF4 was subjected to DLS measurements. Only B and C conditions supplemented with 5 and 10 units/mL of UFH respectively. All the conditions were incubated for 15 min at 25°C. Larger complexes formations were induced upon UFH supplementations.
(DOCX)

S5 Fig The rPF4 zeta potential analysis. (A) Represents the zeta analysis of the elution buffer devoid of any rPF4 proteins serving as negative control. (B) Represents the zeta analysis of 600 μg/mL of rPF4 present in the elution buffer. The secreted rPF4 has a net positive charge as the human derived native PF4.
(DOCX)

S6 Fig SDS-PAGE and Western blotting analysis of the synergistic effects on protein secretion of Glycine, Triton X-100, and IPTG. A total of 36 different conditions with distinct combinations of compounds were analyzed. Mainly the supplementation of low concentrations of Triton X-100 revealed to be the most efficient additive in releasing the trapped rPF4 from the periplasmic compartment.
(DOCX)

S7 Fig The rPF4 secretion mediated by the type II secretory system. The pelB signal sequence directs protein export into the extracellular environment through the SecYEG translocon complex in a process assisted by SecB chaperone. A) Indicates protein secretion before enhancement with chemical supplementation B) indicates secretion in the presence of chemical supplements.
(TIF)

S1 Raw image (PDF)
