# Supplementary material for: High-yield production of recombinant platelet factor 4 by harnessing and honing the gram-negative bacterial secretory apparatus
Source: PLoS One. 2020 May 7;15(5):e0232661. doi: 10.1371/journal.pone.0232661 (PMC7205247; doi:10.1371/journal.pone.0232661)
Supplement: S1 Fig — Protein secretion as a function of bacterial density. Bacterial density follows an increasing trend for the first 20 h, and begins to decline then after, indicating that the total protein secretion has a slower pace than the bacterial growth. The vertical axes, indicates secretion per bacterial density, in μg mL-1 OD-1. (DOCX) [file pone.0232661.s001.docx]

S1 Fig) **the trend of protein secretion over time**

Protein secretion as a function of bacterial density. Bacterial density follows an increasing trend for the first 20 h, and begins to decline then after, indication that total protein secretion has a slower pace than the bacterial growth. The vertical axes, indicates secretion per bacterial density, in terms of micrograms per milliliter per OD.
