# Supplementary material for: High-yield production of recombinant platelet factor 4 by harnessing and honing the gram-negative bacterial secretory apparatus
Source: PLoS One. 2020 May 7;15(5):e0232661. doi: 10.1371/journal.pone.0232661 (PMC7205247; doi:10.1371/journal.pone.0232661)
Supplement: S2 Fig — 100 μg/mL of rPF4 was subjected to DLS measurements. Only C and D conditions were supplemented with 20 units/mL of UFH. All the conditions were incubated for 2 h at 25°C. Approximately 100 nm of large complexes formations were induced between UFH and rPF4 tetramers. (DOCX) [file pone.0232661.s002.docx]

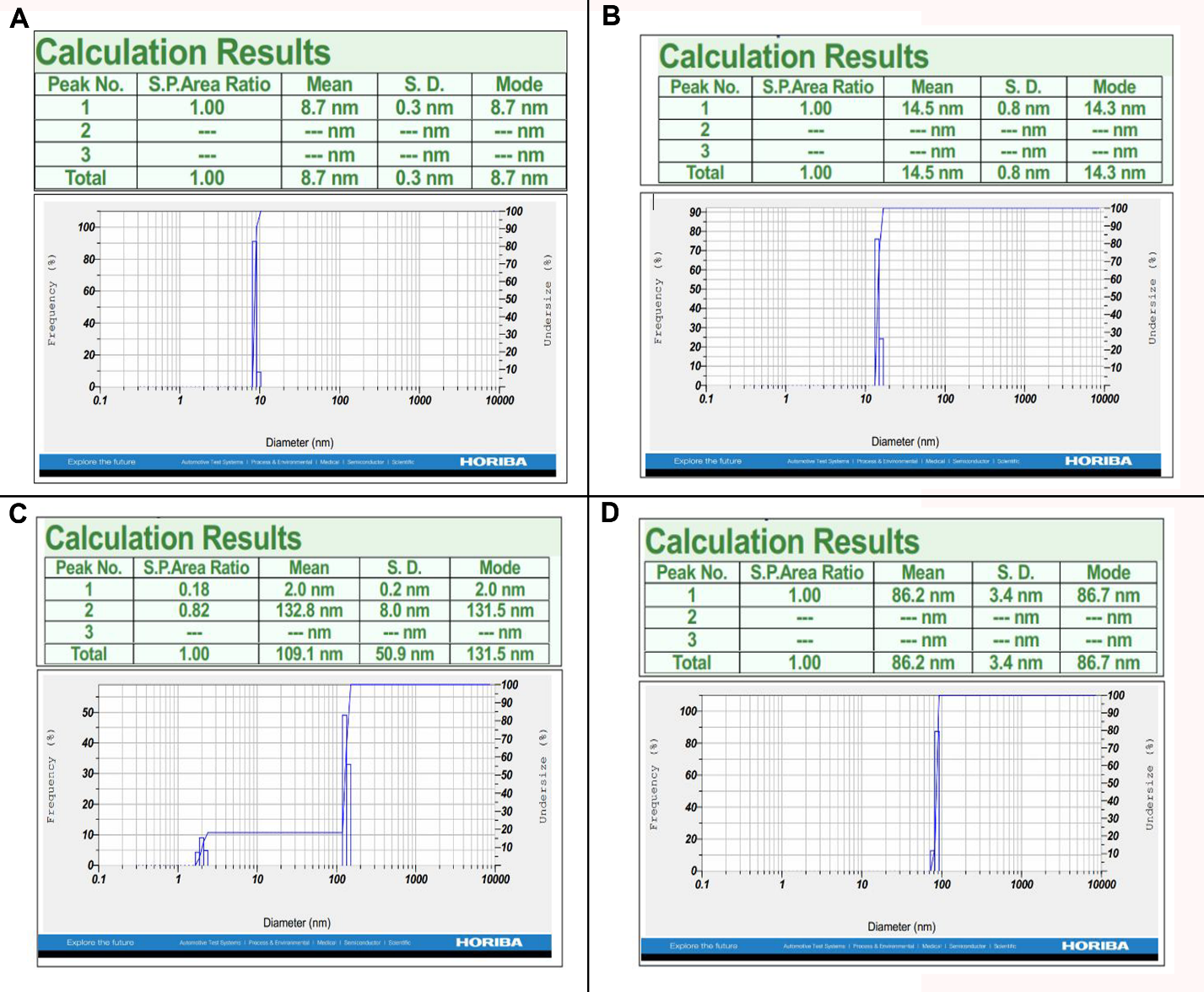


S2 Fig) **100 µg/mL concentration of rPF4 oligomerization analysis.**
100 µg/mL of rPF4 was subjected to DLS measurements. Only C and D conditions were supplemented with 20 units/mL of UFH. All the conditions were incubated for 2 h at 25 °C. Approximately 100 nm of large complexes formations were induced between UFH and rPF4 tetramers.
