# Supplementary material for: High-yield production of recombinant platelet factor 4 by harnessing and honing the gram-negative bacterial secretory apparatus
Source: PLoS One. 2020 May 7;15(5):e0232661. doi: 10.1371/journal.pone.0232661 (PMC7205247; doi:10.1371/journal.pone.0232661)
Supplement: S3 Fig — 200 μg/mL of rPF4 was subjected to DLS measurements. Only C and D conditions were supplemented with 5 units/mL of UFH. All the conditions were incubated overnight. Conditions A and C were stored at 25°C, whereas conditions B and D were stored at 37°C. Very large complexes of 600 nm to 1200nm were formed between UFH and rPF4 tetramers. Storing at 37°C appears to induce a larger complex formation than 25°C. (DOCX) [file pone.0232661.s003.docx]

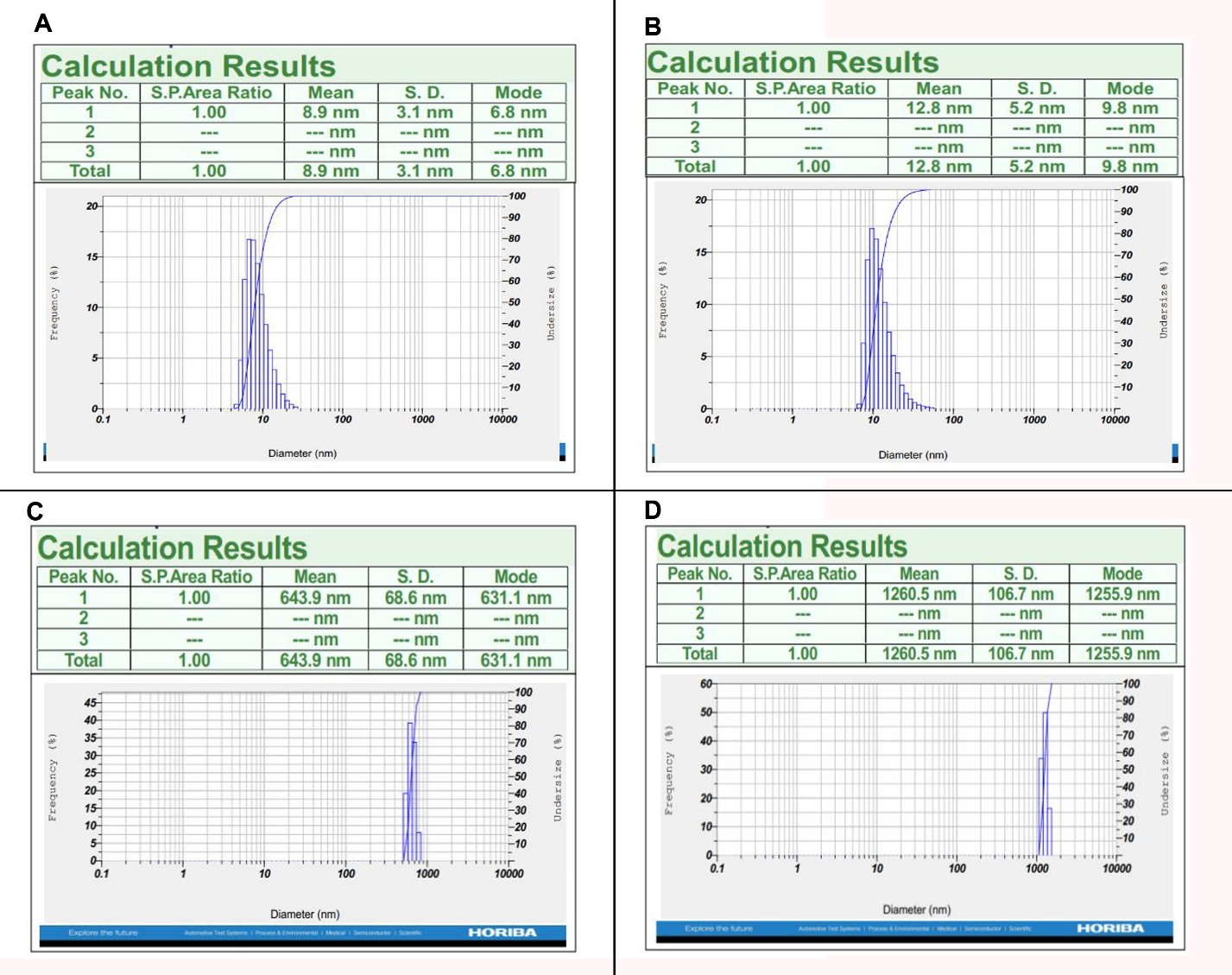


S3 Fig) **200 µg/mL concentration of rPF4 oligomerization analysis.**
200 µg/mL of rPF4 was subjected to DLS measurements. Only C and D conditions supplemented with 5 units/mL of UFH. All the conditions were incubated overnight. Conditions A and C were stored at 25 °C, whereas conditions B and D were stored at 37 °C. Very large complexes of 600 nm to 1200nm were formed between UFH and rPF4 tetramers. Storing at 37 °C appears to induce a larger complex formations than 25 °C.
