# Supplementary material for: High-yield production of recombinant platelet factor 4 by harnessing and honing the gram-negative bacterial secretory apparatus
Source: PLoS One. 2020 May 7;15(5):e0232661. doi: 10.1371/journal.pone.0232661 (PMC7205247; doi:10.1371/journal.pone.0232661)
Supplement: S4 Fig — 400 μg/mL of rPF4 was subjected to DLS measurements. Only B and C conditions supplemented with 5 and 10 units/mL of UFH respectively. All the conditions were incubated for 15 min at 25°C. Larger complexes formations were induced upon UFH supplementations. (DOCX) [file pone.0232661.s004.docx]

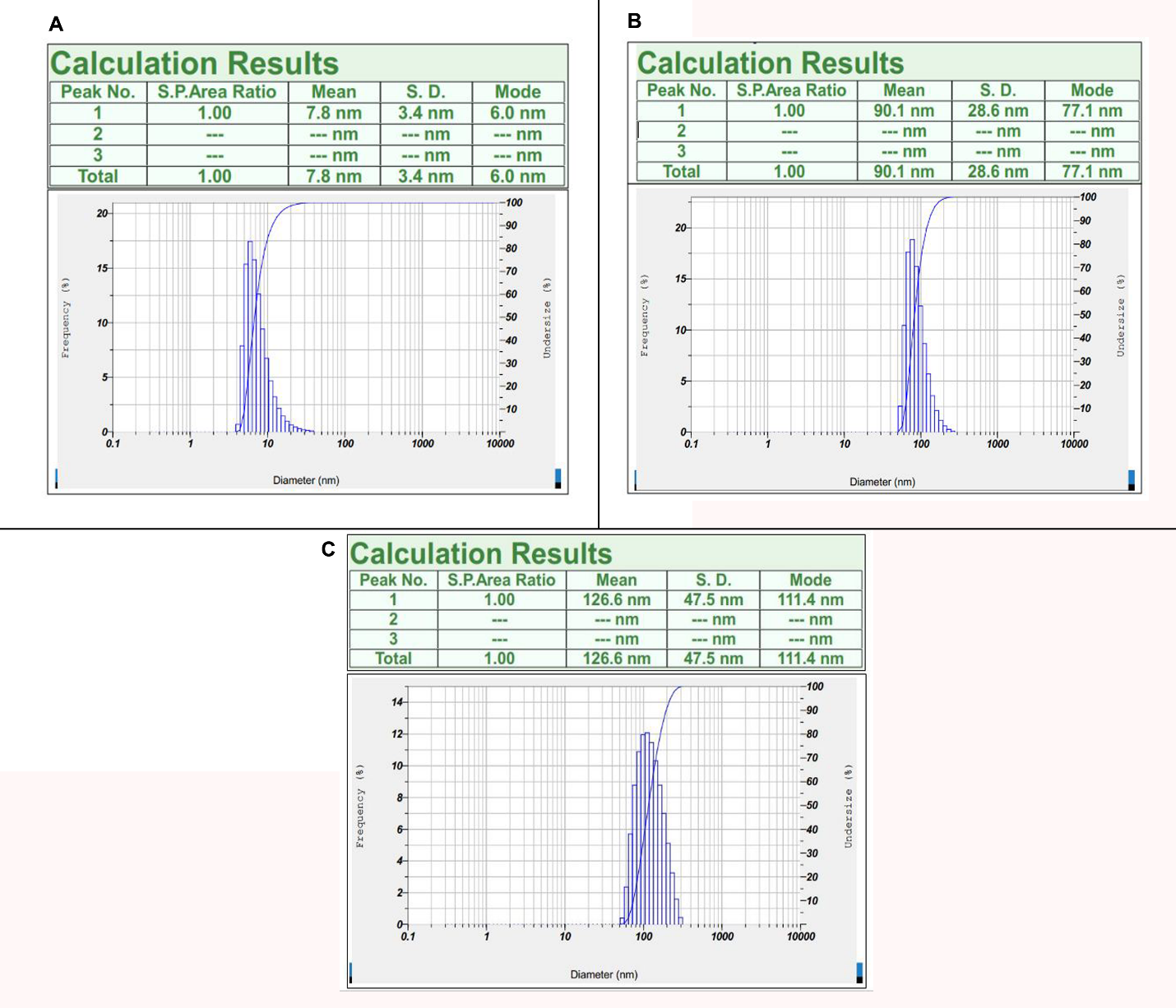


S4 Fig) **400 µg/mL concentration of rPF4 oligomerization analysis**.

400 µg/mL of rPF4 was subjected to DLS measurements. Only B and C conditions supplemented with 5 and 10 units/mL of UFH respectively. All the conditions were incubated for 15 min at 25 °C. Larger complexes formations were induced upon UFH supplementations.
