# Supplementary material for: High-yield production of recombinant platelet factor 4 by harnessing and honing the gram-negative bacterial secretory apparatus
Source: PLoS One. 2020 May 7;15(5):e0232661. doi: 10.1371/journal.pone.0232661 (PMC7205247; doi:10.1371/journal.pone.0232661)
Supplement: S5 Fig — (A) Represents the zeta analysis of the elution buffer devoid of any rPF4 proteins serving as negative control. (B) Represents the zeta analysis of 600 μg/mL of rPF4 present in the elution buffer. The secreted rPF4 has a net positive charge as the human derived native PF4. (DOCX) [file pone.0232661.s005.docx]

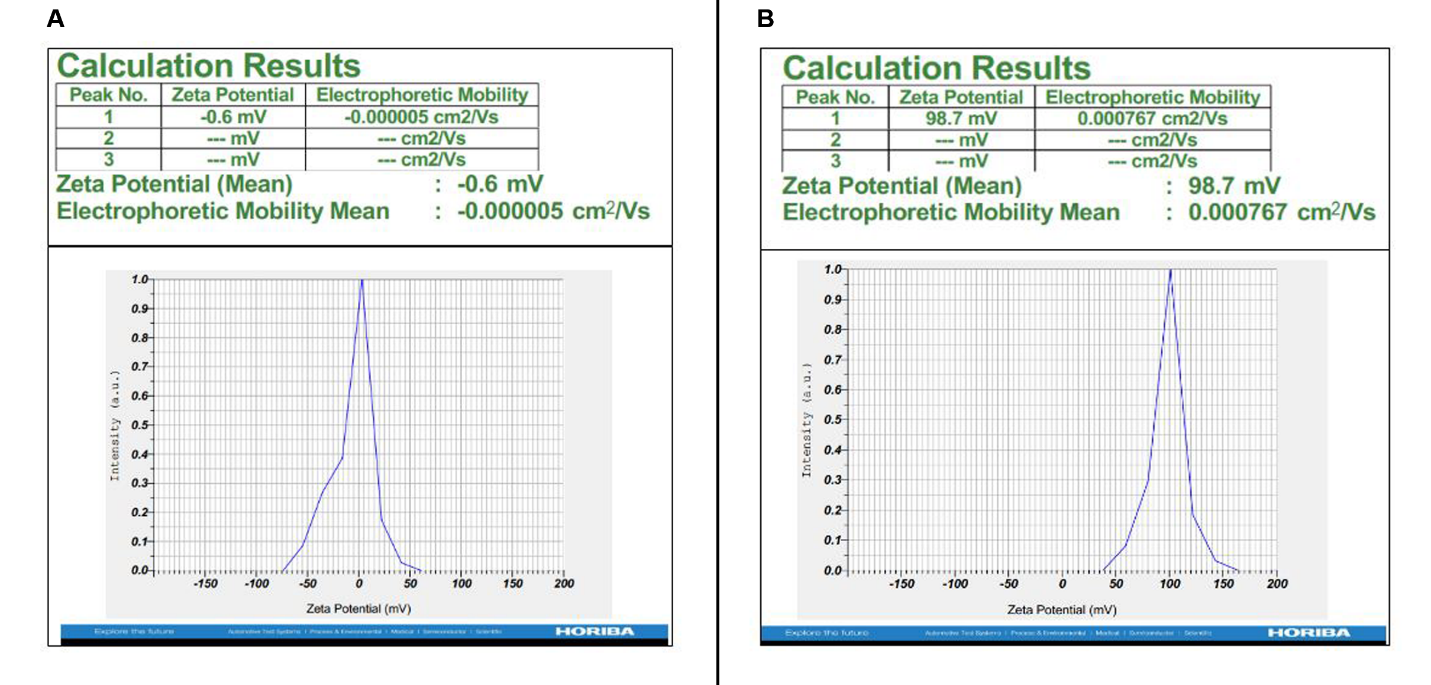


S5 Fig) **rPF4 zeta potential analysis.** (A) Represent the zeta analysis of the elution buffer devoid of any rPF4 proteins serving as negative control. (B) Represents the zeta analysis of 600 µg/mL of rPF4 present in the elution buffer. The recombinant secreted rPF4 similar to the human derived native PF4 harbors a net positive charge.
