# Supplementary material for: High-yield production of recombinant platelet factor 4 by harnessing and honing the gram-negative bacterial secretory apparatus
Source: PLoS One. 2020 May 7;15(5):e0232661. doi: 10.1371/journal.pone.0232661 (PMC7205247; doi:10.1371/journal.pone.0232661)
Supplement: S6 Fig — A total of 36 different conditions with distinct combinations of compounds were analyzed. Mainly the supplementation of low concentrations of Triton X-100 revealed to be the most efficient additive in releasing the trapped rPF4 from the periplasmic compartment. (DOCX) [file pone.0232661.s006.docx]

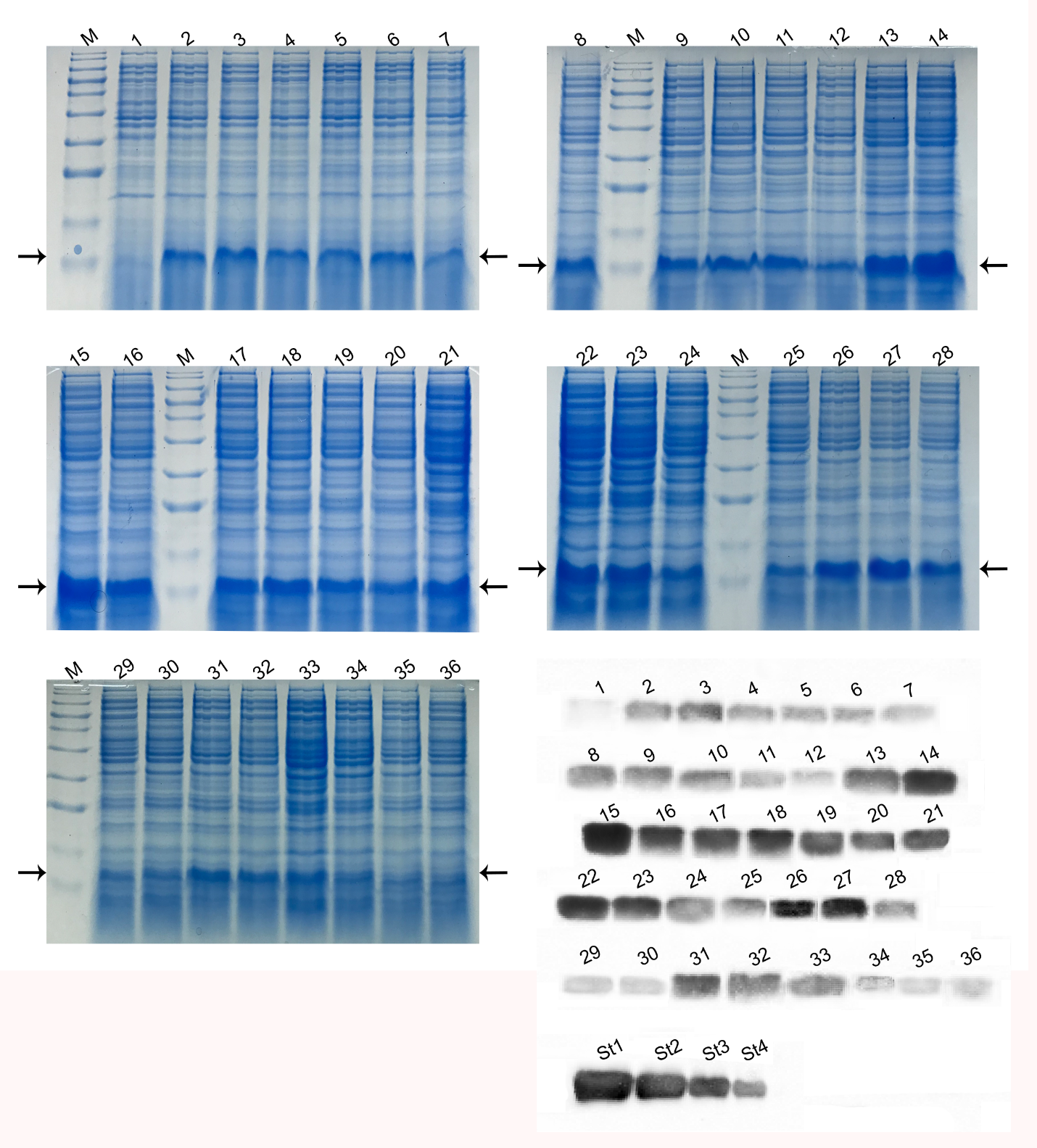


S6 Fig) **SDS-PAGE and Western blotting analysis of the synergistic effect of Glycine, Triton X-100, and IPTG**

A combination of 36 different conditions with distinct combinations of excipients were analyzed. Mainly the supplementation of low concentrations of Triton X-100 revealed to be the most efficient additive in releasing the trapped rPF4 from the periplasmic compartment.
