# Supplementary figures and images for: High-yield production of recombinant platelet factor 4 by harnessing and honing the gram-negative bacterial secretory apparatus
Source: PLoS One. 2020 May 7;15(5):e0232661. doi: 10.1371/journal.pone.0232661 (PMC7205247; doi:10.1371/journal.pone.0232661)

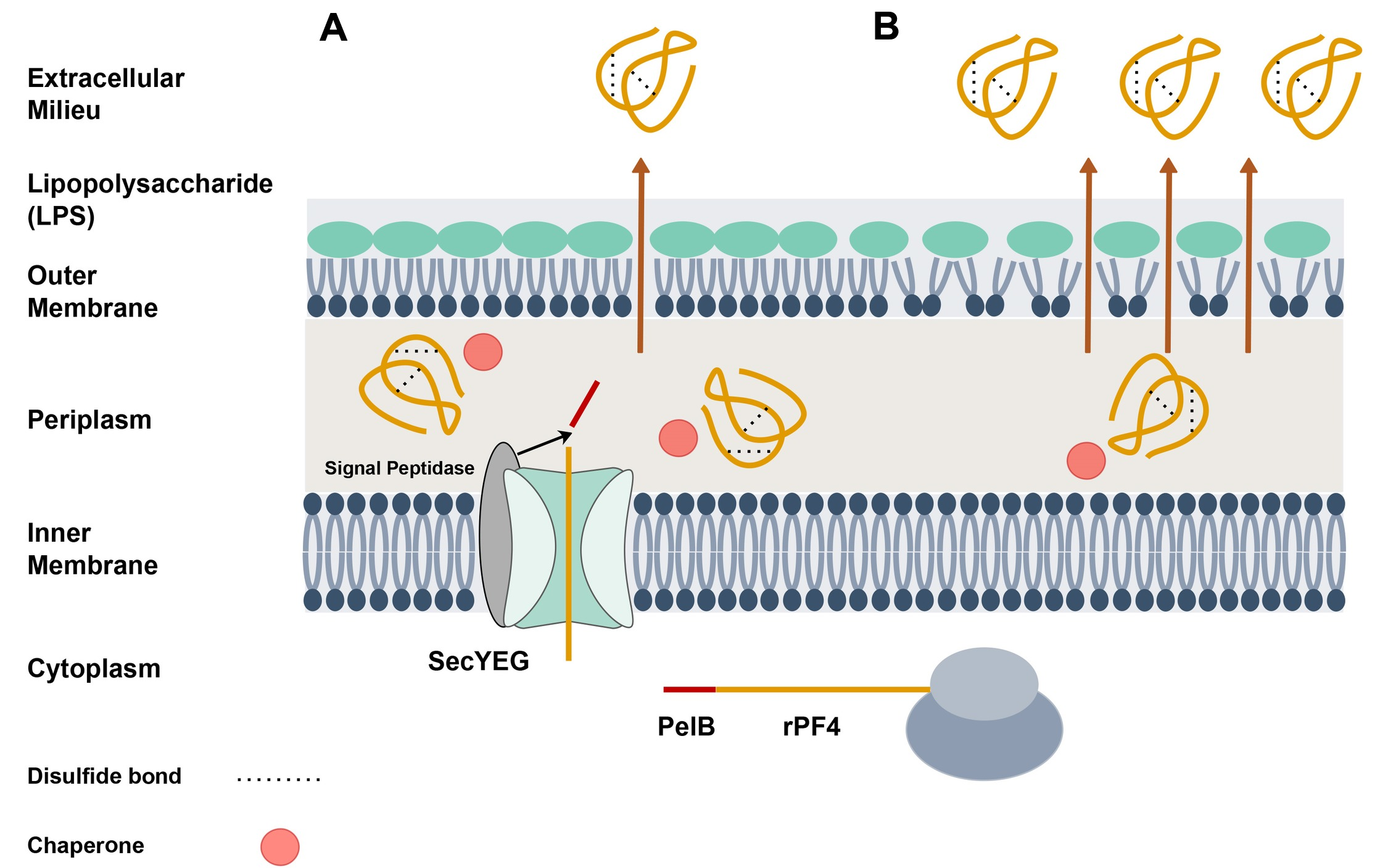

Supplement: S7 Fig — The pelB signal sequence directs protein export into the extracellular environment through the SecYEG translocon complex in a process assisted by SecB chaperone. A) Indicates protein secretion before enhancement with chemical supplementation B) indicates secretion in the presence of chemical supplements. (TIF) [file pone.0232661.s007.tif]

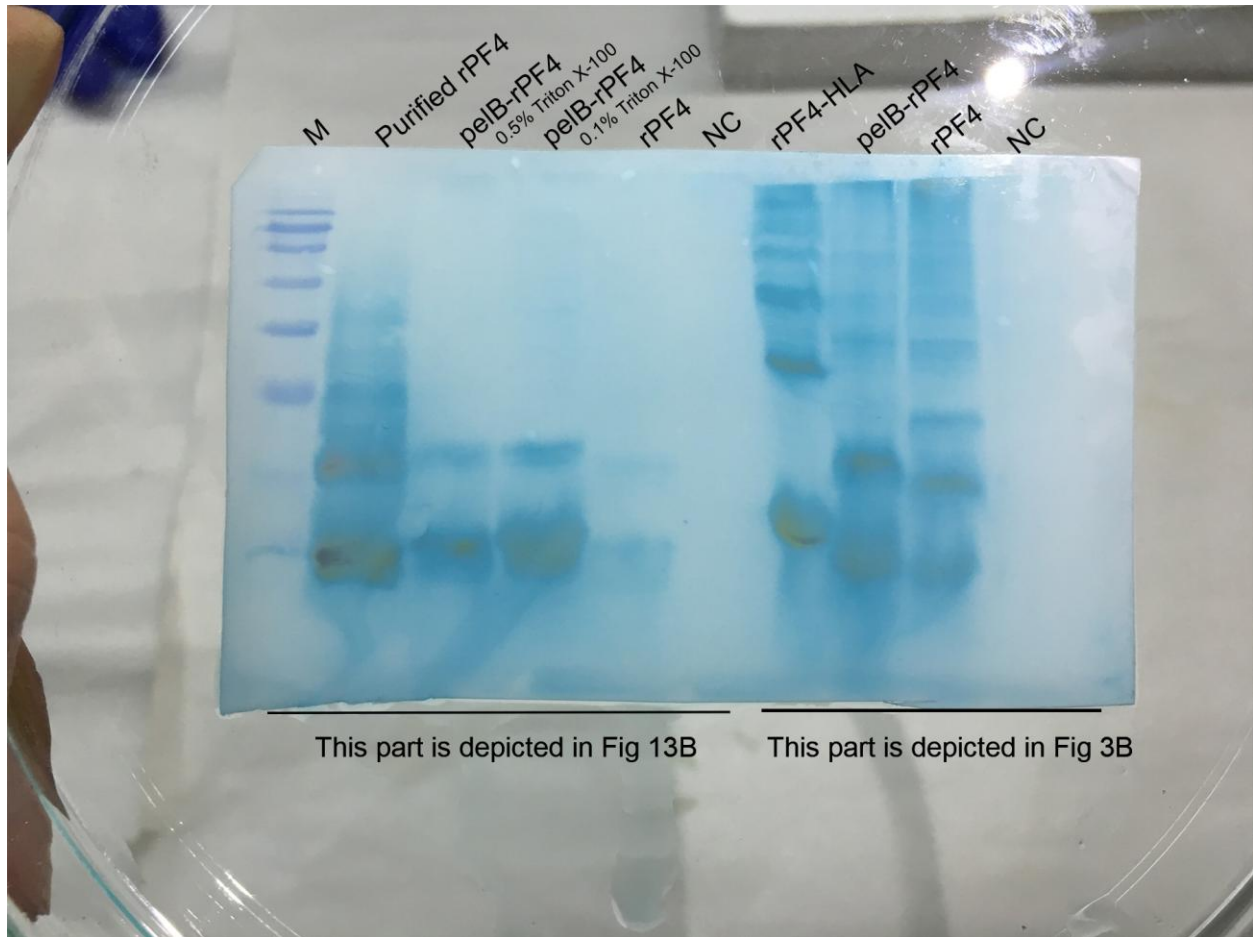

**Fig 13B and Fig 3B**

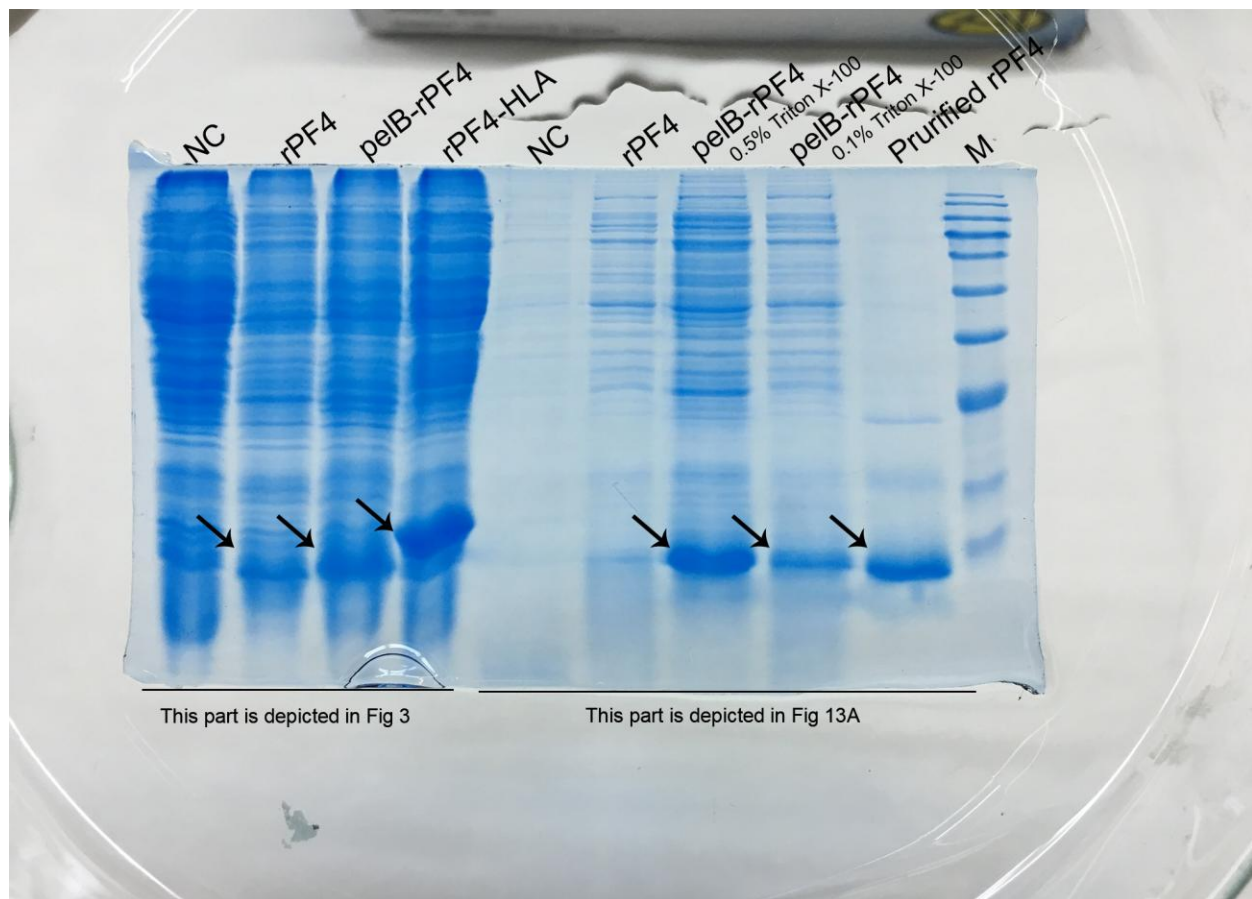

**Fig 3 and Fig 13A**

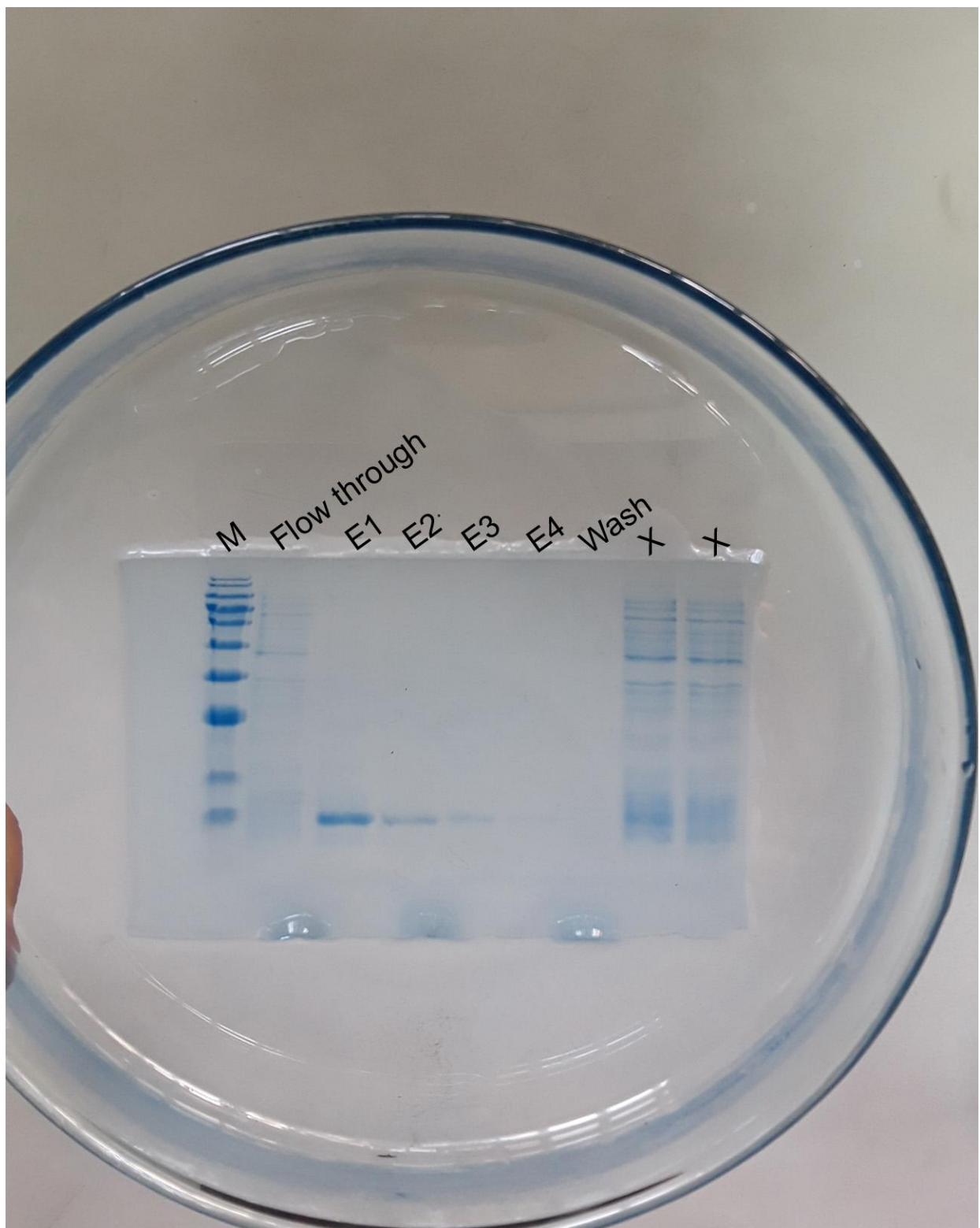

**Fig 4**

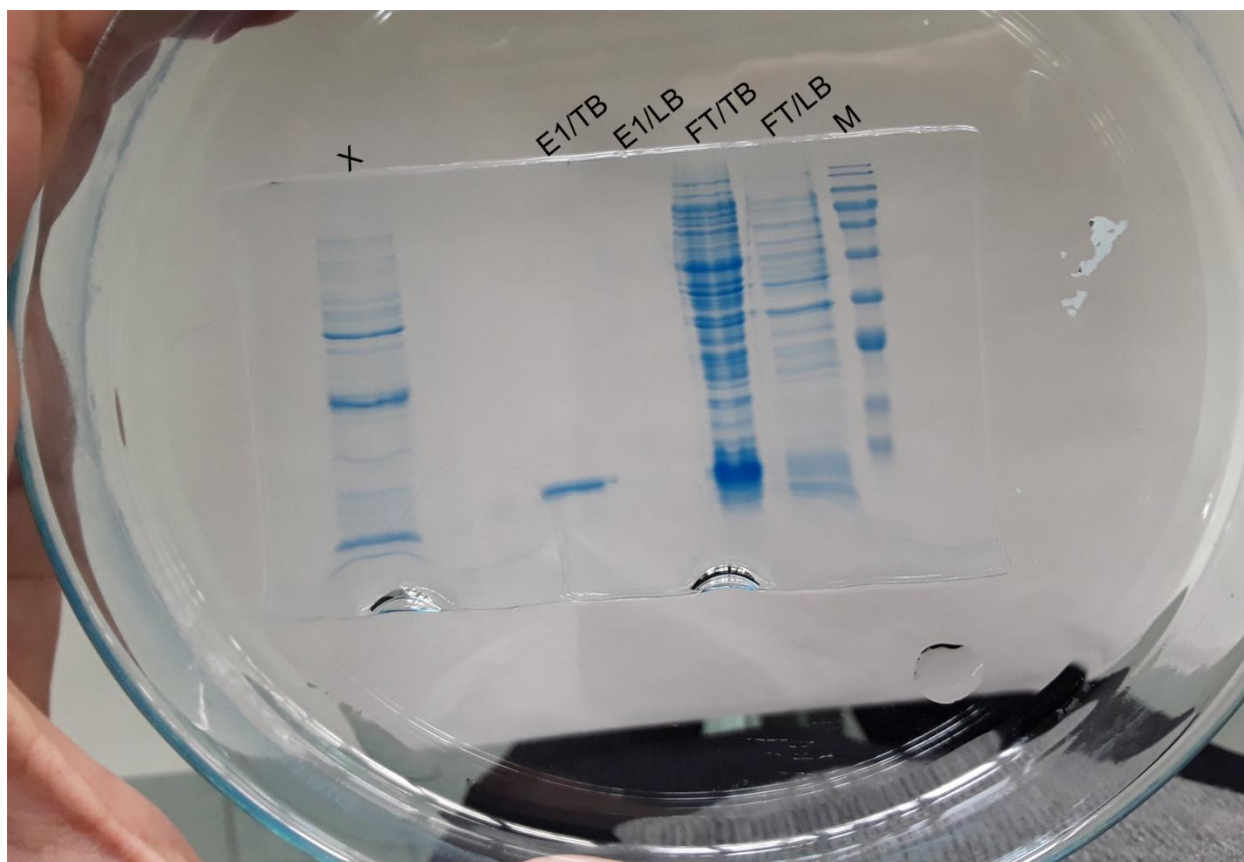

**Fig 5A**

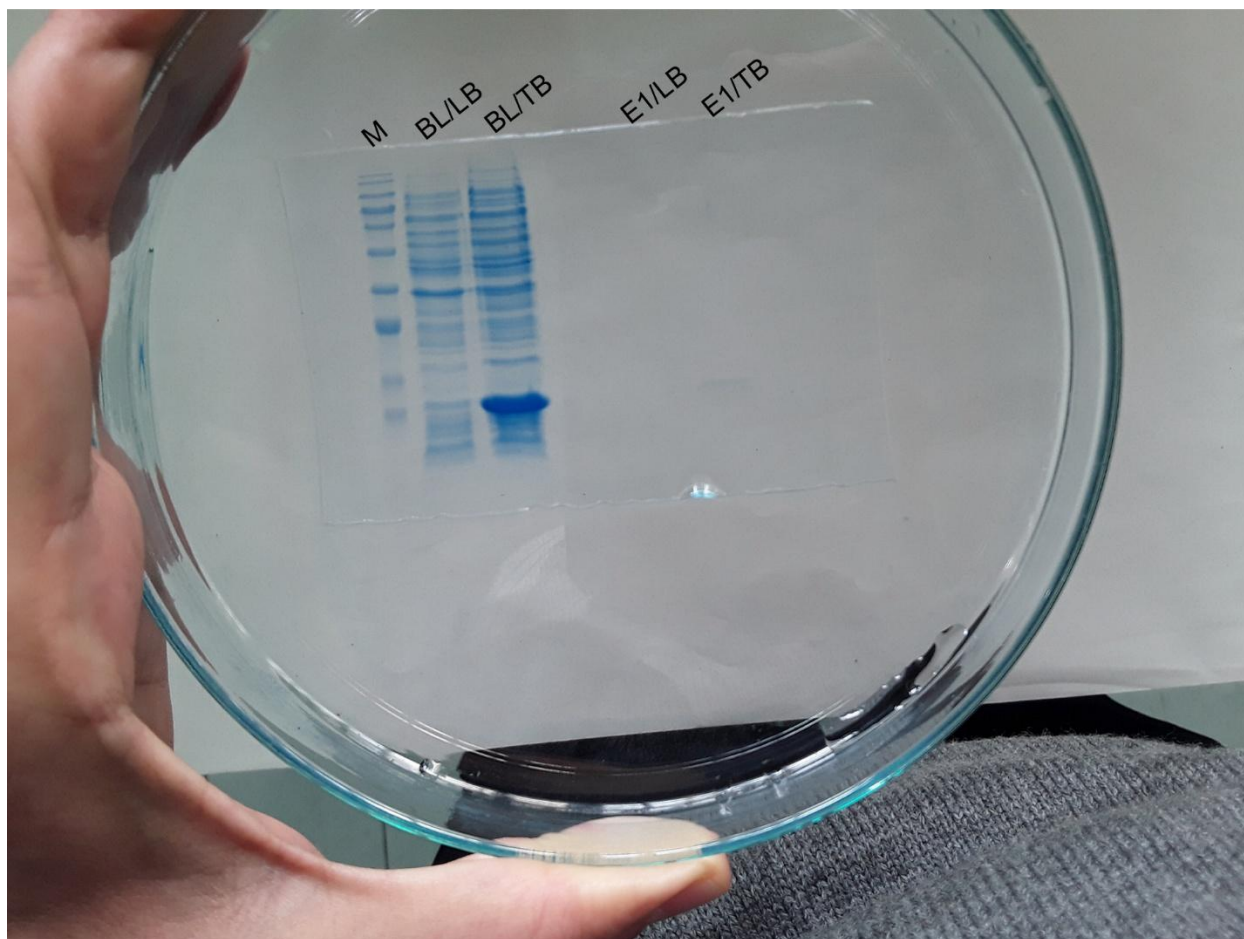

**Fig 5B**

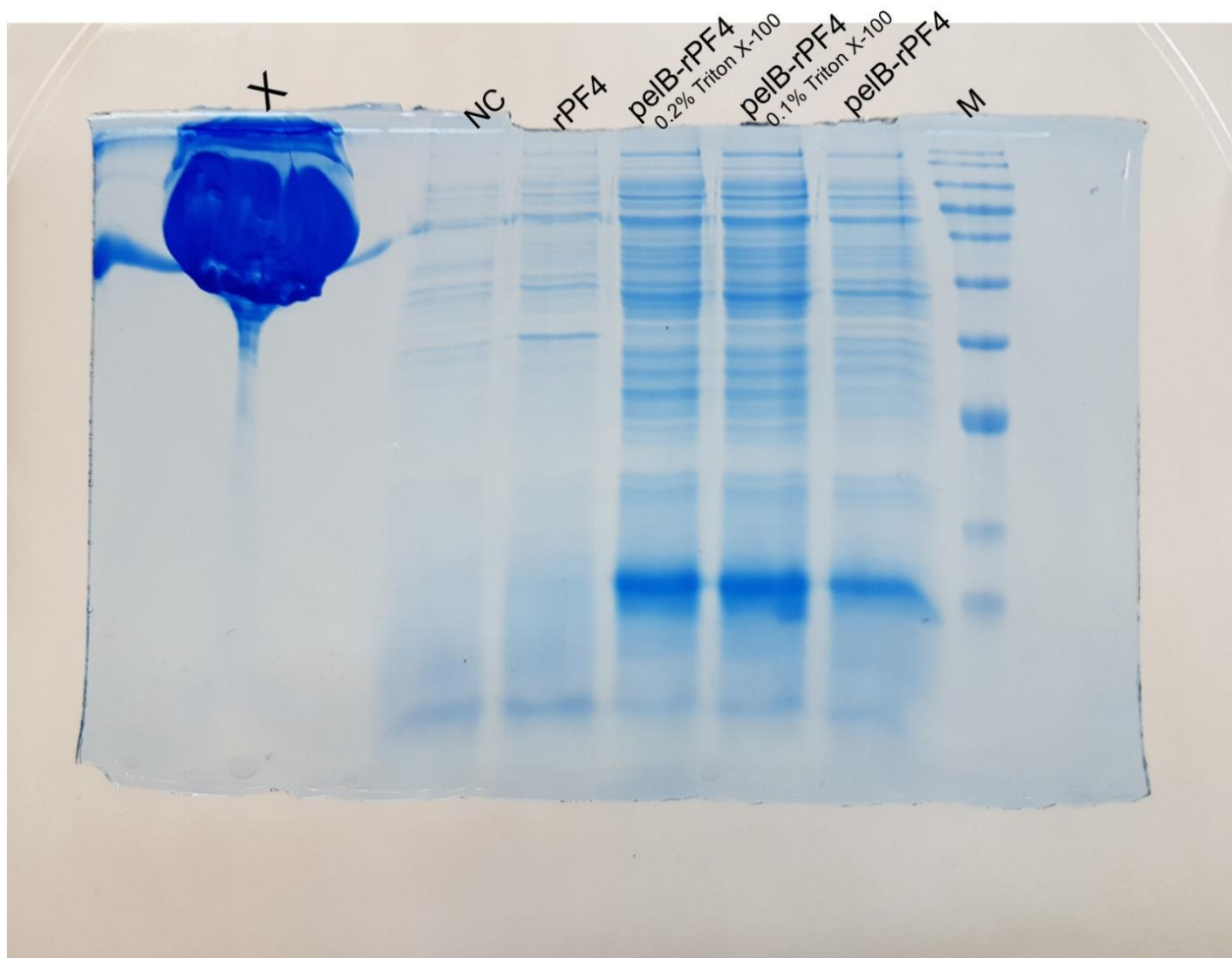

**Fig 6A**

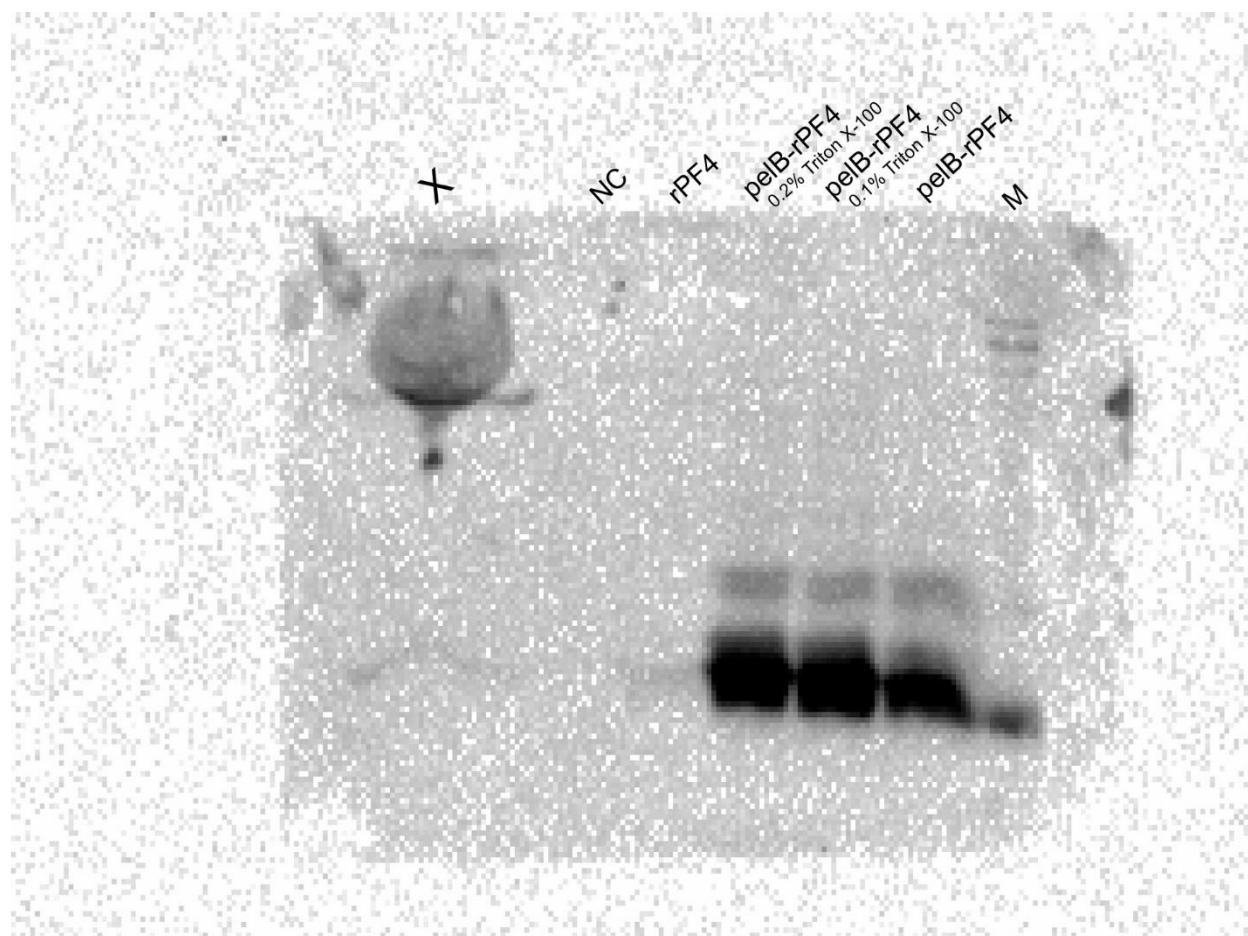

**Fig 6B**

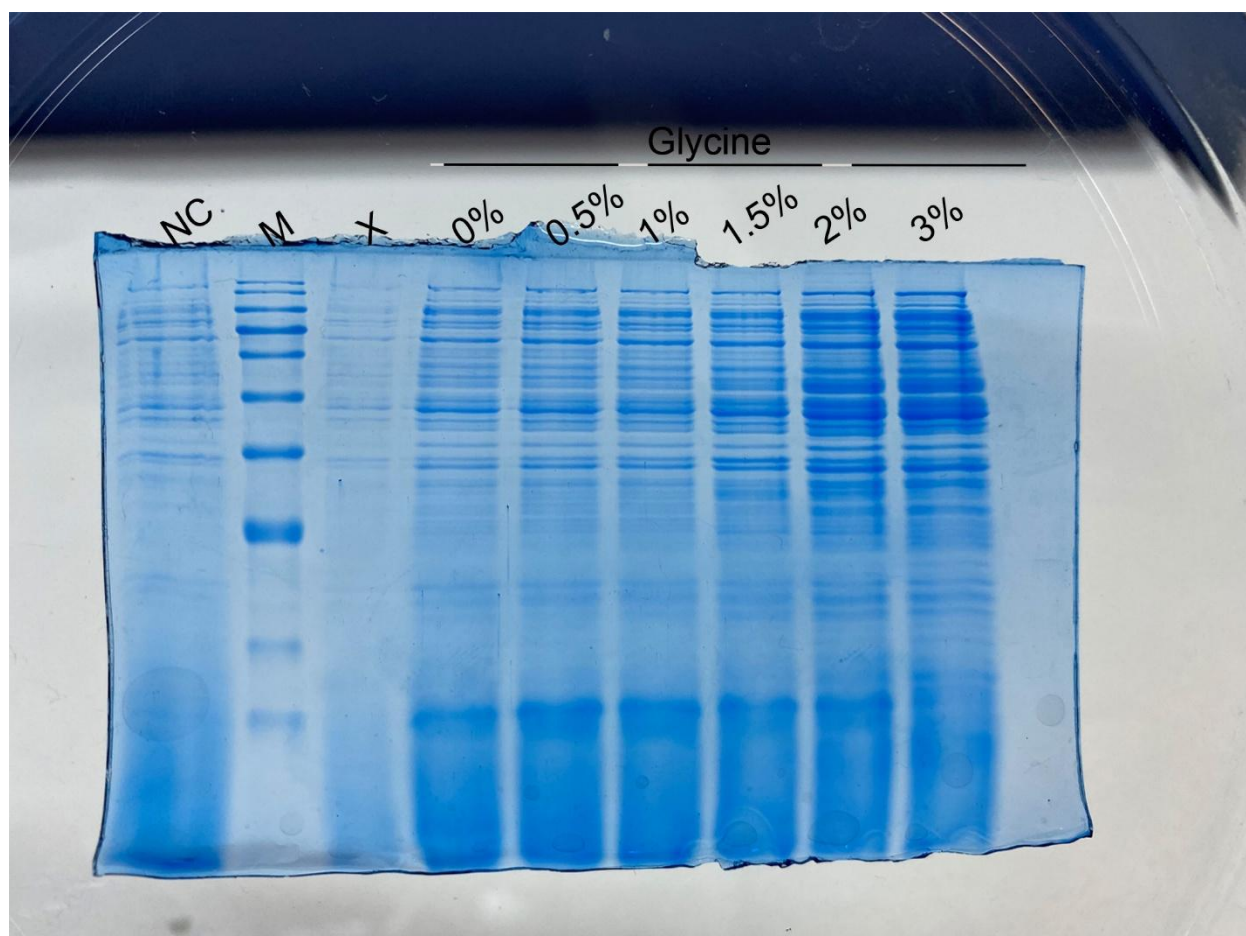

**Fig 7A**

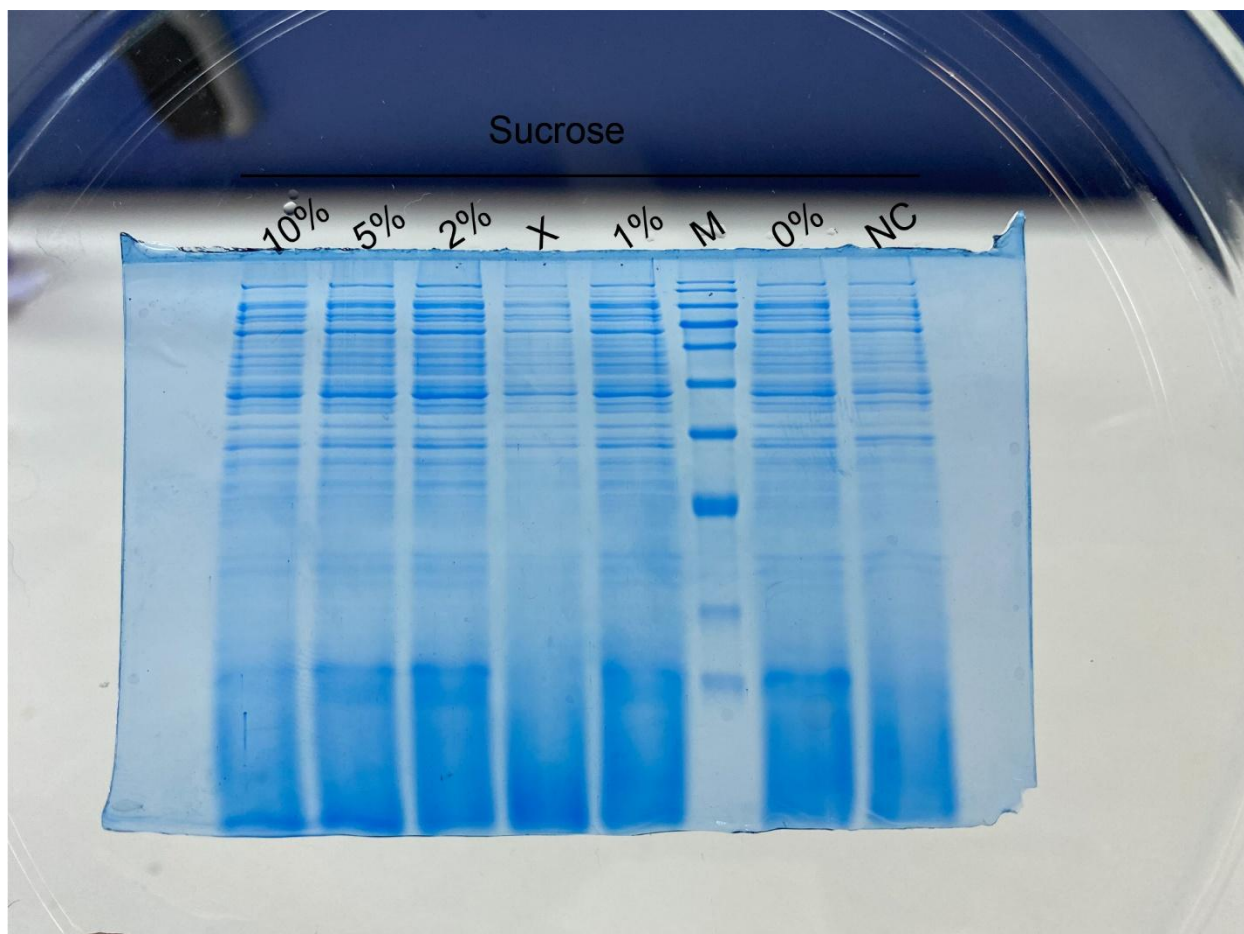

**Fig 7B**

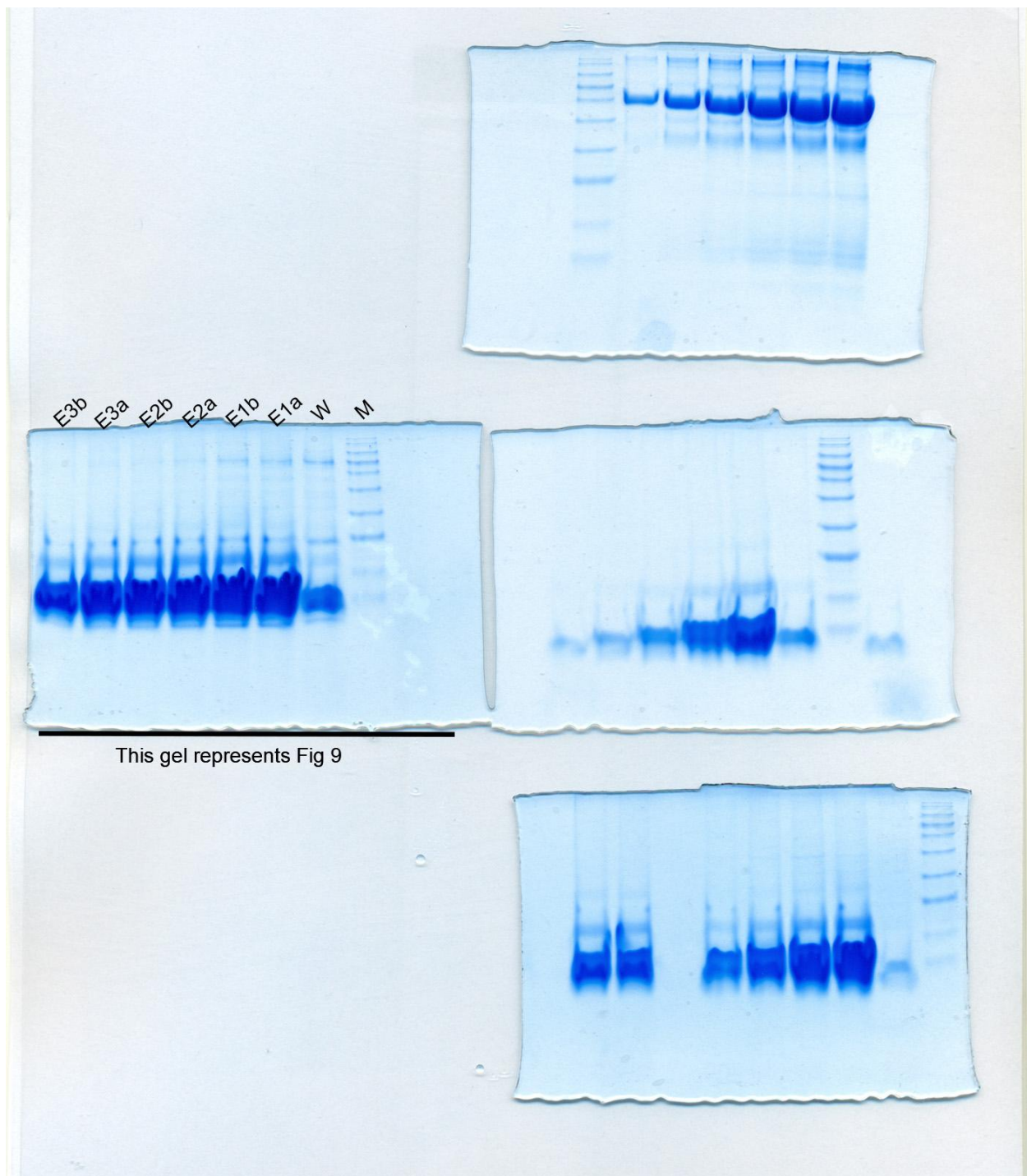

This gel represents Fig 9

**Fig 9**

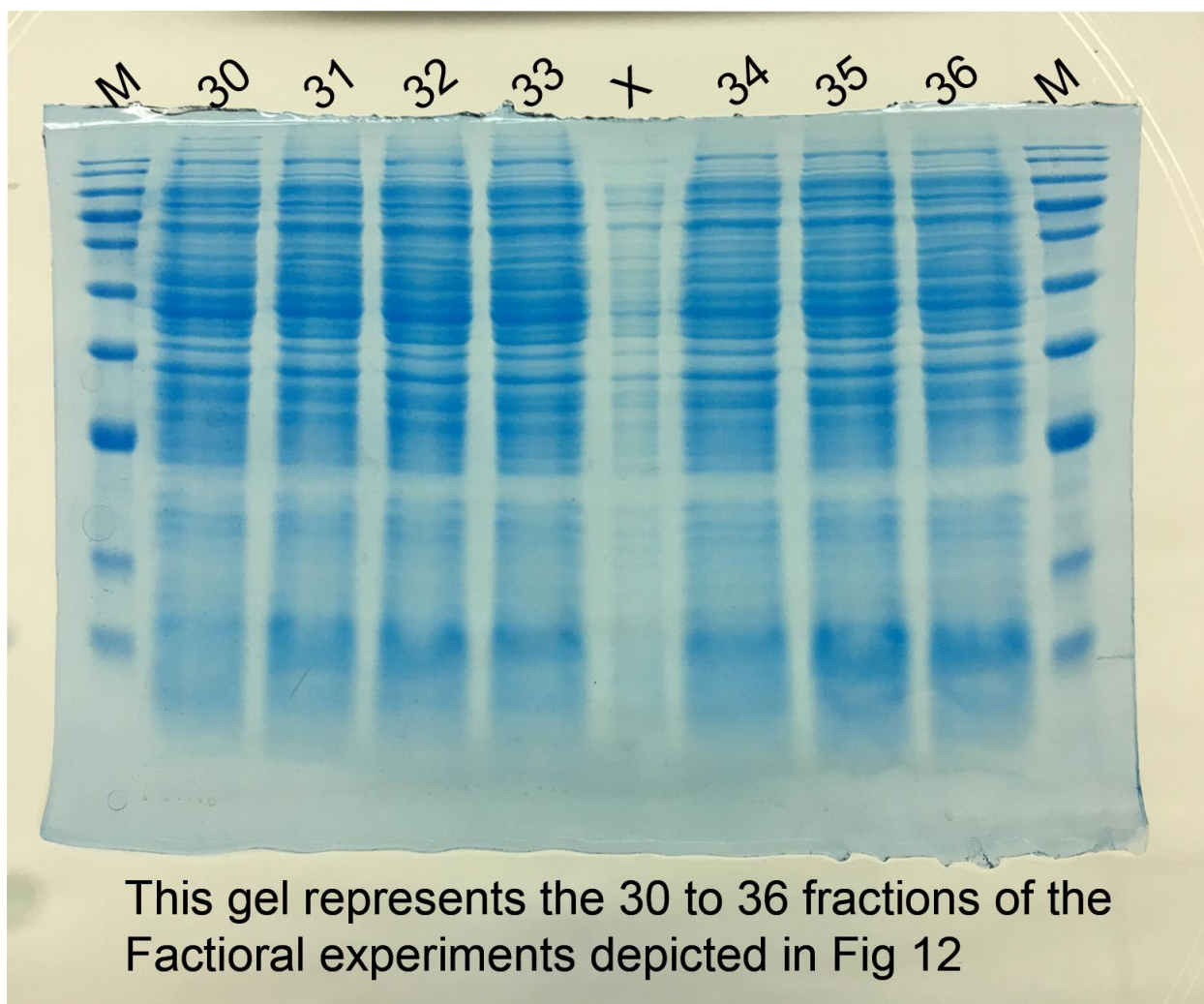

**Fig 12**

Supplement: S1 Raw image — (PDF) [file pone.0232661.s008.pdf]
